# Oxygen Enhanced Optoacoustic Tomography (OE-OT) Reveals Vascular Dynamics in Murine Models of Prostate Cancer

**DOI:** 10.7150/thno.19841

**Published:** 2017-07-08

**Authors:** Michal R Tomaszewski, Isabel Quiros Gonzalez, James PB O'Connor, Oshaani Abeyakoon, Geoff JM Parker, Kaye J Williams, Fiona J Gilbert, Sarah E Bohndiek

**Affiliations:** 1 Department of Physics, University of Cambridge, U.K.;; 2 Cancer Research UK Cambridge Institute, University of Cambridge, U.K.;; 3 Institute of Cancer Sciences, University of Manchester, U.K.;; 4 Department of Radiology, The Christie NHS Foundation Trust, U.K.;; 5 Department of Radiology, University of Cambridge, U.K.;; 6 Centre for Imaging Sciences, University of Manchester, U.K.;; 7 Bioxydyn Limited, Manchester, U.K.;; 8 Manchester Pharmacy School, University of Manchester, U.K.

**Keywords:** optoacoustic, imaging, oxygenation, angiogenesis, hypoxia.

## Abstract

Poor oxygenation of solid tumours has been linked with resistance to chemo- and radio-therapy and poor patient outcomes, hence non-invasive imaging of oxygen supply and demand in tumours could improve disease staging and therapeutic monitoring. Optoacoustic tomography (OT) is an emerging clinical imaging modality that provides static images of endogenous haemoglobin concentration and oxygenation. Here, we demonstrate oxygen enhanced (OE)-OT, exploiting an oxygen gas challenge to visualise the spatiotemporal heterogeneity of tumour vascular function. We show that tracking oxygenation dynamics using OE-OT reveals significant differences between two prostate cancer models in nude mice with markedly different vascular function (PC3 & LNCaP), which appear identical in static OT. LNCaP tumours showed a spatially heterogeneous response within and between tumours, with a substantial but slow response to the gas challenge, aligned with *ex vivo *analysis, which revealed a generally perfused and viable tumour with marked areas of haemorrhage. PC3 tumours had a lower fraction of responding pixels compared to LNCaP with a high disparity between rim and core response. While the PC3 core showed little or no dynamic response, the rim showed a rapid change, consistent with our *ex vivo *findings of hypoxic and necrotic core tissue surrounded by a rim of mature and perfused vasculature. OE-OT metrics are shown to be highly repeatable and correlate directly on a per-tumour basis to tumour vessel function assessed *ex vivo. *OE-OT provides a non-invasive approach to reveal the complex dynamics of tumour vessel perfusion, permeability and vasoactivity in real time. Our findings indicate that OE-OT holds potential for application in prostate cancer patients, to improve delineation of aggressive and indolent disease as well as in patient stratification for chemo- and radio-therapy.

## Introduction

Angiogenesis is required for delivery of oxygen and nutrients to the proliferating tumour volume beyond ~1mm^3^ 1. The chaotic and tortuous nature of the resulting vasculature leads to substantial intra-tumoural heterogeneity in vascular density and function^2^, respectively leading to diffusion- and perfusion-limited hypoxia^3^. These hypoxic stresses increase tumour heterogeneity^4^ and result in poor prognosis^5^,^6^ through increased chemo- and radio-resistance^2^,^7^, particularly in cancers of hormone sensitive tissues such as the prostate^6^. Non-invasive imaging of oxygen supply and demand in prostate cancer could therefore improve diagnosis and staging, by delineating aggressive from indolent disease, and assist with therapeutic decision making, by identifying tumours with reduced vascular function, which limits delivery of chemotherapy and decreases efficacy of radiotherapy.

Magnetic resonance imaging (MRI)-based approaches such as blood oxygen level dependent (BOLD) and oxygen enhanced (OE) MRI^8^ have been shown to correlate with tissue oxygen partial pressure (pO_2_) and histological markers of hypoxia^9^,^10^. Combined T_2_* and T_1_ contrast^11^ as well as breathing gas challenges^12^,^13^ have previously been applied in prostate cancer, and the advantages of oxygen contrast in comparison to exogenous contrast enhancement in functional studies has been evaluated^14^. However, these functional MRI techniques suffer from intrinsically low sensitivity, poor spatial resolution, and in case of BOLD, signals that may be confounded by several biological factors^15^,^16^. Positron emission tomography (PET) agents for hypoxia visualisation are also available^17^, but these measurements are limited by the inherently low spatial resolution of PET and risks related to radioisotope administration. Hence, while MRI and PET approaches hold some promise, there remains an unmet clinical need for validated imaging biomarkers of vascular density and function, as well as vascular heterogeneity, that can be measured cost effectively at high spatial and temporal resolution^18^.

Optoacoustic tomography (OT) is an emerging imaging modality^19^,^20^ that is currently in clinical trials^20^,^21^ and has the potential to fulfil this unmet need. OT reveals the distribution of tissue optical absorption coefficient in real time with a spatial resolution of ~190μm at 3cm penetration depth^22^. Since the absorption spectra of oxy- and deoxy-haemoglobin are distinct, acquiring OT data at multiple wavelengths makes it possible to derive images of haemoglobin concentration and oxygenation. OT has recently been shown qualitatively to: visualise and quantify microvasculature in 3D with high resolution^23^,^24^; monitor vascular development during tumour growth^25^-^27^; detect response to vascular targeted therapies^28^,^29^ and predict radiotherapy response^30^. OT has also been paired with contrast enhanced ultrasound^31^,^32^ to discriminate cellular necrosis from blood lakes^32^. While promising, these studies take only a static readout and quantify mean tumour values hence lack quantitative insight into vascular dynamics or the spatial heterogeneity of vascular function within or between tumours. We hypothesized that by studying the spatiotemporal response of the tumour vasculature to an oxygen challenge using OT, we could gain greater insight into tumour vessel dynamics. Here, we demonstrate that oxygen enhanced (OE) OT provides a highly repeatable and robust readout of tumour vascular function, studying both spatial heterogeneity and evolution during tumour growth, which is relevant in the context of prostate cancer staging. We present for the first time a significant per tumour correlation between the tumour OE-OT dynamics and histopathological measures including an *ex vivo* Hoechst 33342 dye uptake, which serves to model delivery of a small molecule agent. We find that OE-OT can delineate two prostate cancer xenograft models with markedly different vascular characteristics that appear identical in terms of their static OT haemoglobin oxygenation measurement: the androgen-independent, aggressive, poorly differentiated and highly metastatic PC3^33^; and the androgen-sensitive, slow-growing, highly differentiated, haemorrhagic LNCaP^34^. We provide the first biological validation of OE-OT as a technique for dynamic visualization of the spatial heterogeneity in tumour vascular function.

## Methods

### Animal Experiments

All animal procedures were conducted in accordance with project (70-8214) and personal license (IDCC385D3) issued under the United Kingdom Animals (Scientific Procedures) Act, 1986 and were approved locally under compliance form number CFSB0671. Subcutaneous prostate tumours were established in male BALB/c nude mice (Charles River): Two prostate adenocarcinoma cell lines (obtained from CRUK Cambridge Institute biorepository), suspended in PBS and Matrigel (Corning) in equal parts, were implanted subcutaneously in two different cohorts: PC3 (n=33 mice, 1.5x10^6^ cells, up to 200µL in right flank) and LNCaP (n=15 mice, 1.5x10^6^ cells, up to 100µL in both flanks). Authentication using Genemapper ID v3.2.1 (Genetica) by STR Genotyping (1/2015) showed 94% match in both cases. Tumour growth was monitored regularly by callipers (Figure S1) and by imaging at three time points; mice were sacrificed at intermediate time-points and at the study end point, before tumours reached 10% body weight. Volume matching within tumour type was performed as follows: the first imaging session was performed when tumours reached > 30mm^3^; the intermediate imaging session was performed at the mid-point for growth; and the final imaging session was taken at the limits of ethical approval, based on tumour volume or animal welfare. Exclusion criteria for mice are detailed in the Supplementary Methods.

### Optoacoustic Imaging

For optoacoustic imaging, a MultiSpectral Optoacoustic Tomography (MSOT) inVision 256-TF small animal imaging system (iThera Medical GmbH) was used^35^. Briefly, a tunable optical parametric oscillator (OPO) pumped by an Nd:YAG laser provides excitation pulses with a duration of 9ns at wavelengths from 660nm to 1200nm at a repetition rate of 10Hz with a wavelength tuning speed of 10ms and a peak pulse energy of 90mJ at 720nm. Ten arms of a fiber bundle provide uniform illumination of a ring-shaped light strip of approximately 8mm width. For ultrasound detection, 256 toroidally focused ultrasound transducers with a center frequency of 5MHz (60% bandwidth), organized in a concave array of 270 degree angular coverage and a radius of curvature of 4cm, are used.

**Figure 1:**
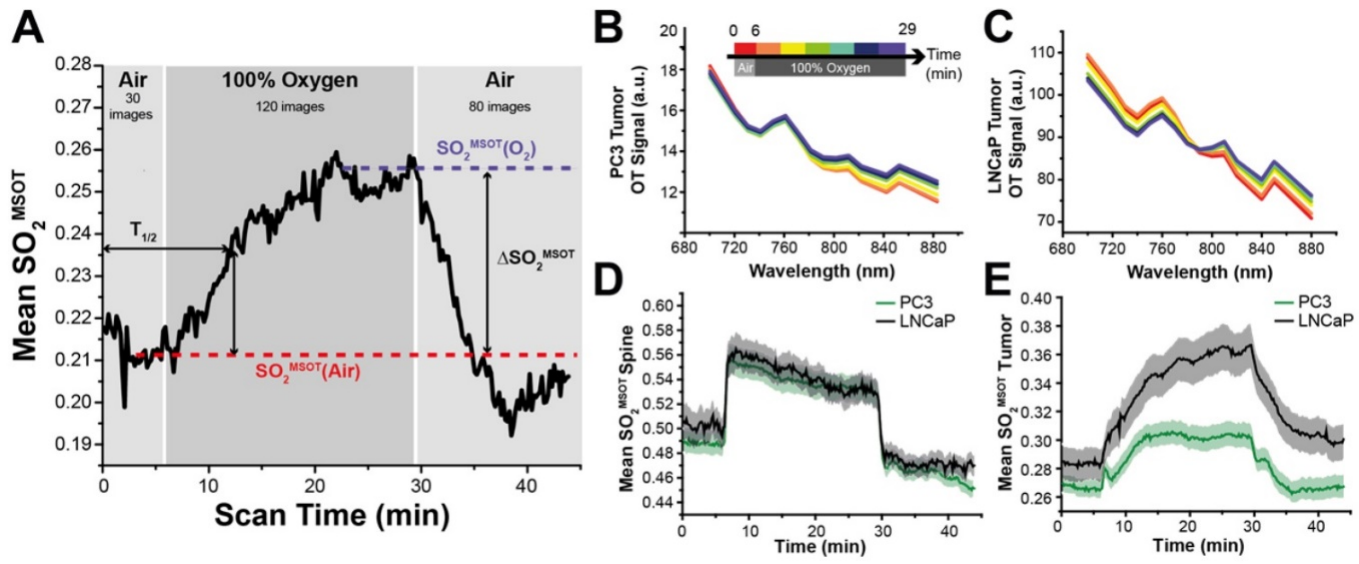
**Temporal evolution of the oxygen enhanced optoacoustic tomography (OE-OT) signal.** An example kinetic curve taken from a PC3 tumour subject to an oxygen gas challenge is shown in (A). The metrics extracted from the OE-OT data illustrated in (A) are: baseline SO_2_^MSOT^ values before [SO_2_^MSOT^(Air)] and after the oxygen challenge [SO_2_^MSOT^(O_2_)]; amplitude of signal change (ΔSO_2_^MSOT^); and time to half-maximum (T_1/2_). Averaged spectral changes for PC3 and LNCaP tumours are shown in (B) and (C) respectively, while kinetic profiles of SO_2_^MSOT^ averaged across all healthy tissue ROIs and tumour xenograft ROIs are shown in (D) and (E) respectively, with standard error of the mean indicated by the shaded envelope (n_t_=53, n_b_=30 PC3, n_t_=35, n_b_=17 LNCaP).

Mice were prepared according to our standard operating procedure^36^. Briefly, mice were anaesthetised using <3% isoflurane and placed in a custom animal holder (iThera Medical), wrapped in a thin polyethylene membrane, with ultrasound gel (Aquasonic Clear, Parker Labs) used to couple the skin to the membrane. The holder was then placed within the MSOT system and immersed in degassed water maintained at 36°C. Mice were allowed to stabilise for 15 minutes within the system prior to initialisation of the scan and their respiratory rate was then maintained in the range 70-80bpm with ~1.8% isoflurane concentration for the entire scan. Manipulation of the breathing gas between medical air (21% oxygen) and pure oxygen (100% oxygen) was performed manually using separate flow meters according to the schedule in Figure 1A. When tumours were established on both flanks, the imaging field of view was placed within the larger tumour at the time of the first imaging session, unless both tumours were in the same plane. Images were acquired through the centre of the tumour using 15 wavelengths between 700nm and 880nm and an average of 7 pulses per wavelength; a single slice acquisition was 11.5s in duration. A subset of the PC3 cohort (n=8) were imaged twice, with 24h between the scans to allow for full recovery from anaesthesia, to assess the repeatability of the oxygen challenge data.

The small molecule (616Da) dye Hoechst 33342 (Thermo Fisher Scientific) extravasates into tumour tissue, giving an indication of vascular permeability and perfusion^37^, important for the assessment of the vascular function^38^,^39^ and simulating the uptake of a small molecule therapeutic agent. Mice were injected intravenously in the tail vein with 120μL of Hoechst 33342 for a 20g mouse at 2.5mg/ml in PBS (yielding 15mg/kg) two minutes before sacrifice^40^ by cervical dislocation. Immediately after sacrifice, tumours were excised.

### Magnetic Resonance Imaging

Mice bearing PC3 (n=7) and LNCaP (n=3) tumours underwent magnetic resonance imaging (MRI) on a 9.4T Agilent MRI system running VnmrJ 3.1, using a quadrature transmit/receive millipede volume coil (Agilent, inc) of 38mm inner diameter after the OE-OT scan. The same anaesthesia and oxygen challenge protocol as for optoacoustic imaging experiments was used. The mouse was cannulated before being placed in the holder and its temperature was monitored using a rectal probe, stabilized to 37^O^C using air heating system. BOLD MRI data were then acquired (see Supplementary Methods for details). After the oxygen challenge the animals were injected with the Gadolinium-based Gadovist agent (Bayer, 200µmol/kg) through the intravenous cannula. DCE-MRI data were then acquired (see Supplementary Methods for details). The mice then underwent optoacoustic imaging and the results were compared on a per-tumour basis.

### Oxylite Measurements

An invasive Oxylite pO_2_ probe (Oxford Optronics Ltd.) was used to assess changes in local oxygen partial pressure in response to the oxygen challenge in PC3 tumour bearing mice (n=6) as detailed in the Supplementary Methods.

### Histopathologic Tumour Analysis

Tumours from the two cohorts were either mounted on a cork base using OCT solution (VWR Chemicals) and snap frozen in an isopentane bath cooled on dry ice for fluorescence analysis or divided into two (parallel to the imaging plane) with the other half being fixed in neutral buffered 10% formalin (24h) for haematoxylin and eosin (H&E) staining. Formalin fixed, paraffin embedded (FFPE) tumours were sectioned with 3µm thickness at 4 equally spaced levels. Additionally, immunohistochemistry was performed. Consecutive adjacent sections from 5 PC3 and 5 LNCaP tumours in the centre were stained with CD31 (anti-mouse, BD Biosciences, 553370) and alpha smooth muscle actin (ASMA) (anti-mouse, Abcam, ab5694) to indicate vessel density and smooth muscle coverage respectively. Further sections were stained with CAIX (anti-human, BioScience Slovakia, AB1001) to indicate hypoxic regions.

Frozen blocks were sectioned with 6µm thickness at 4 equally spaced levels. To indicate vascular density, immunofluorescence staining of CD31 was performed using anti-mouse CD31 primary antibody (555370, BD Biosciences) and an AlexaFluor647 conjugated anti-rat secondary antibody (A21472, Invitrogen Molecular Probes). Sections were mounted in a DAPI-free mounting media (S36936 SlowFade, Life Technologies) and scanned using a confocal microscope with laser wavelengths of 350nm (for Hoechst) and 633nm (Leica TCS SP5, Leica Microsystems). In each section, up to 10 fields of view (1.02mm x 1.02mm) were randomly selected and imaged using both colour channels.

### Image and Statistical Analysis

All image analysis was performed in MATLAB (Mathworks) using custom software unless otherwise stated.

Optoacoustic image reconstruction was performed using an acoustic backprojection algorithm (iThera Medical) with an electrical impulse response correction, to account for the frequency dependent sensitivity profile of the transducers^41^. Images were reconstructed with a pixel size of 75μm x 75μm, which is approximately equal to half of the in-plane resolution of the InVision 256-TF.

Regions of interest (ROIs) were drawn manually around the tumours (excluding the skin) and a healthy tissue region around the spine in the 800nm (isosbestic) image of the first frame of the oxygen challenge data (Figure S2). The tumour size is taken as the area of the ROI, which correlates directly to calliper measurements (r=0.81, p<0.0001; n=22). The optoacoustic spectrum was averaged across all pixels in the ROIs and a pseudoinverse matrix inversion (pinv function in MATLAB 2014A) was used for spectral unmixing of the relative weights of oxy- [HbO_2_] and deoxy-haemoglobin [Hb] (example images shown in Figure S2). Optoacoustic tomography is only able to accurately resolve absolute SO_2_ if the recorded signal is directly related to the absorbed optical energy distribution, which requires knowledge of the light fluence distribution, system response and Grueneisen parameter^42^. We therefore denote the oxygenation metric derived in this study as an apparent metric, SO_2_^MSOT^ rather than absolute SO_2_. SO_2_^MSOT^ was computed as the ratio of oxygenated [HbO_2_] to total haemoglobin [HbO_2_+Hb]. Rim-core segmentation was performed by successively shrinking the tumour ROI inwards in 0.5mm steps. The two outer regions are referred to as the 'Rim', while the remaining part of the tumour is the 'Core' in the bulk analysis, based on qualitative evaluation of our histopathology data. A more detailed depth dependent analysis was performed in addition on large tumours (where at least 5 separate regions exist) with the outer 4 regions of 0.5mm thickness analysed individually as contributing to the 'Rim' and the remaining as the 'Core'.

Kinetic SO_2_^MSOT^ profiles for each tumour were smoothed using a moving average and analysed as shown in Figure 1A. Extracted metrics of baseline SO_2_^MSOT^ values before [SO_2_^MSOT^(Air)] and after oxygen challenge [SO_2_^MSOT^(O_2_)] informed on the equilibrium status of the tumour. OE-OT metrics included: amplitude of signal change (ΔSO_2_^MSOT^); time to half-maximum (T_1/2_); and the 'Responding Fraction' (RF) of the tumour. RF was quantified by reconstructing the images on an array of 225μm x 225μm pixels, for which spectral unmixing was performed on a per-pixel basis. A pixel was classified as 'responding' if the ΔSO_2_^MSOT^ exceeded 0.03 (insensitive to threshold value, see Figure S3) and RF was taken as the ratio of responding to total number of pixels.

MRI data for T_1_ and T_2_* were fitted pixel-by-pixel to a monoexponential function to produce relaxation maps. The mean T_2_* value across all tumour pixels was extracted from BOLD data. DCE analysis was performed by applying the model of Tofts and Kermode^43^ to extract the mean K^trans^, the transfer coefficient of contrast agent into the tissue, describing tumour perfusion and permeability.

Histopathological analysis of FFPE sections was performed on images scanned at 20x magnification using an Aperio ScanScope (Leica Biosystem) scanner to determine tumour necrotic fraction and ASMA vessel coverage. Confocal fluorescence microscopy images were used to quantify the vascular characteristics based on Hoechst uptake and CD31 staining. Details of the quantification procedures can be found in the Supplementary Methods. Following image processing and identification of blood vessels^44^, the proportion of the derived total vessel area to total image area was quantified as 'CD31 Vascular Area Fraction' (CD31 VF). An estimate of the fraction of tumour receiving oxygen from the vessels (Perfused Area - CD31 PA) was calculated by expanding the identified vessels radially by 100μm, which is the approximate oxygen diffusion distance 45,46. Example images from each processing step are shown in Figure S4. The amount of Hoechst uptake (Hoechst Intensity - HI) was quantified as the average fluorescent signal intensity in the imaged tumour area.

Errors are shown as the standard error on the mean, unless otherwise stated. All statistical analyses were performed in GraphPad Prism 6 (Graphpad Software Inc.). Pearson rank test was performed to assess correlations between the optoacoustic tomography metrics and MRI or histopathology parameters. Paired two-tailed t-test compared the same tumour type; unpaired two-tailed t-test assuming unequal variances compared between tumour types. The significance of SO_2_^MSOT^ changes in the time-series was assessed by a two-way un-matched ANOVA test. p<0.05 was considered significant. For correlations with histology, only the last OE-OT scan before sacrifice was used. For comparisons of OE-OT metrics between the tumour types all technical replicates (imaging sessions) not excluded from the study are included. The number of all technical (imaging sessions) and biological (number of tumours) replicates is quoted with each result, denoted with n_t_ and n_b _respectively. Where a single n quoted, n is the number of biological replicates and no technical replicates were used.

## Results

### OE-OT delineates the vascular function of two prostate cancer xenografts

Application of the oxygen challenge resulted in clearly visible changes in the multispectral optoacoustic tomography (MSOT) derived oxygen saturation (SO_2_^MSOT^, Figure 1A). During the challenge, the measured absorption spectrum of both PC3 (Figure 1B) and LNCaP (Figure 1C) tumours increased above 800 nm, where oxyhaemoglobin dominates. SO_2_^MSOT^ of the healthy tissue around the spine shows a sharp, step function-like response to the oxygen challenge (Figure 1D) convolved with a slight downward drift (related to anaesthesia effects in healthy tissue^36^). The tumours, however, show a significantly lower baseline SO_2_^MSOT^(Air) (PC3: 0.266±0.007 tumour vs. 0.488±0.010 healthy, n_t_=53, n_b_=30, p<0.0001; LNCaP: 0.286±0.011 tumour vs. 0.512±0.019 healthy, n_t_=35, n_b_=17 p<0.0001) and a more gradual SO_2_^MSOT^ response with a >4-fold longer time to half-maximum (Figure 1D,E; PC3: T_1/2_=300±23s tumour vs. 74±3s healthy, n_t_=44, n_b_=28, p<0.0001; LNCaP: T_1/2_=353±30s tumour vs. 84±5s healthy, n_t_=31, n_b_=15, p<0.0001). A similar shape of response is observed in independent pO_2_ measurements (Figure S5) and in the raw oxy- and deoxy-haemoglobin data (Figure S6A,B). Interestingly, the total haemoglobin (THb) signal shows evidence of a change in the haemoglobin concentration in both tumour (Figure S6C) and healthy (Figure S6D) vasculature at the time points of the gas switch, which could arise due to the vasoconstrictive effect of oxygen breathing. Neither SO_2_^MSOT^ nor THb in the tumour were observed to change over a similar time course without the oxygen challenge (Figure S6E, F).

### OE-OT metrics show high repeatability

We assessed the repeatability of our metrics by performing a second OE-OT study 24 hours after the first in a subset of PC3 tumours (Figure S7A). Significant correlations between test and retest (Figure S7B) were found for SO_2_^MSOT^(O_2_) (r=0.76, p<0.01; n=8), Responding Fraction RF (r=0.96, p<0.001; n=8) and ΔSO_2_^MSOT^ (r=0.89, p<0.01; n=8), indicating good repeatability of these OE-OT metrics. T_1/2_ could not always be robustly computed so was considered the least repeatable OE-OT metric. The static SO_2_^MSOT^ metrics of THb (r=0.18, p=0.70; n=7) and SO_2_^MSOT^(Air) (r=0.64, p=0.09; n=8), show little or no correlation between test and retest, indicating poor repeatability for these metrics. Dynamic OE-OT metrics may therefore be more robust than static OT for longitudinal studies.

### OE-OT provides spatiotemporal insight not available using static OT

**Figure 2:**
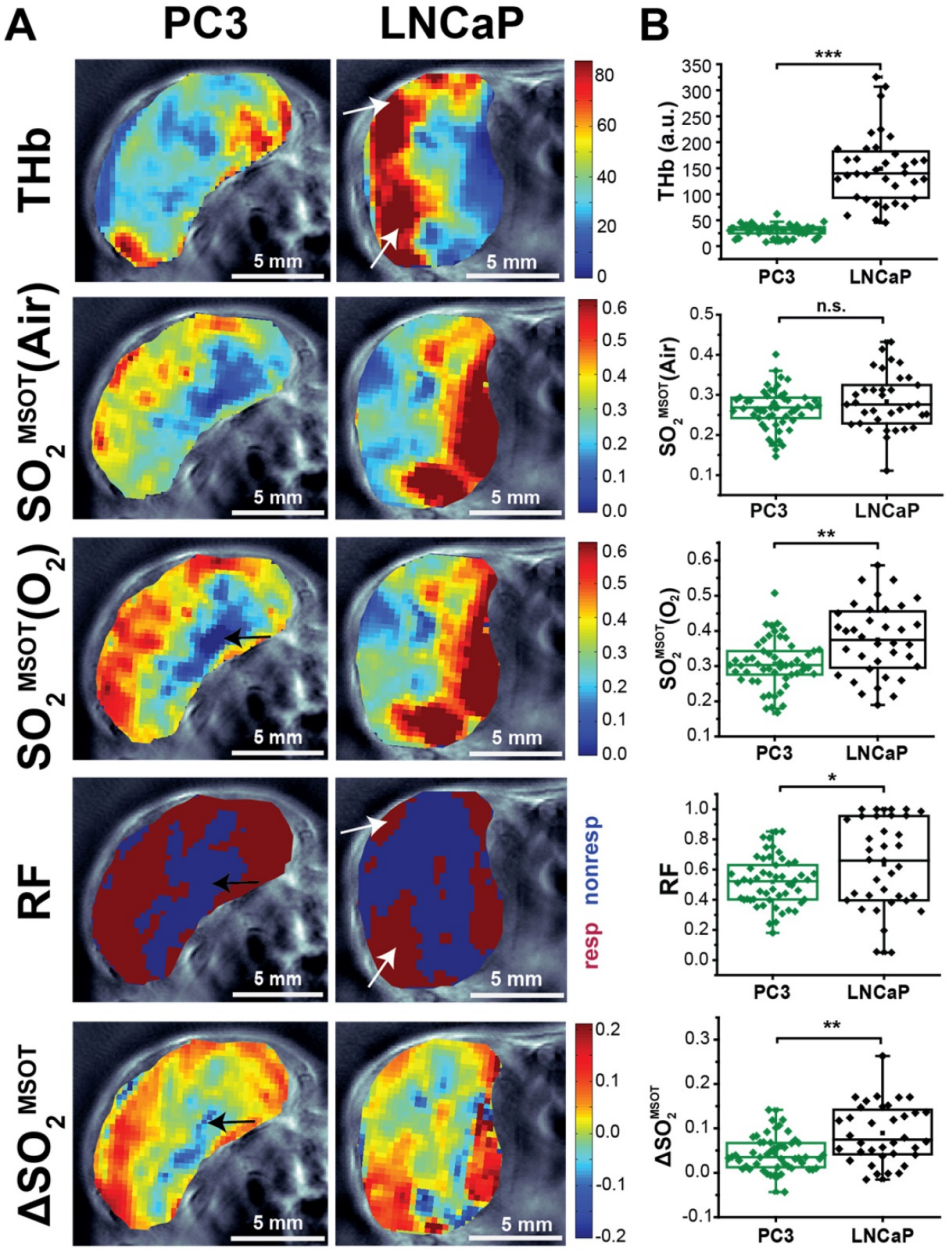
** OT and OE-OT signals are spatially heterogeneous in PC3 and LNCaP tumours. **(A) Representative images of PC3 (left) and LNCaP (right) tumours showing the spatial distribution of total haemoglobin (THb), baseline oxygen saturation before the oxygen challenge [SO_2_^MSOT^(Air)] and the OE-OT metrics of: oxygen saturation after the oxygen challenge [SO_2_^MSOT^(O_2_)]; responding fraction (RF), which indicates the distribution of responding and non-responding pixels; and the amplitude of the signal change in responding pixels (ΔSO_2_^MSOT^). All OE-OT metrics are significantly different between the two tumour types, highlighting vascular differences not visible in the static picture. Black arrows indicate responding pixels in the PC3 tumour core, white arrows indicate response of haemorrhagic regions in LNCaP tumours. (B) Quantification of the mean tumour values for each optoacoustic metric studied. Each point represents the mean of the given metric over the tumour in a technical replicate. * p<0.05, ** p<0.01, *** p<0.001 by unpaired two-tailed t-test (unequal variances), n_t_=53, n_b_=30 PC3, n_t_=35, n_b_=17 LNCaP. Box between 25^th^ and 75^th^ percentile, line at median.

In order to examine the ability of OE-OT to provide greater insight into tumour vascular dynamics and heterogeneity, we first compared the spatial distribution (Figure 2A) and mean values (Figure 2B) of static OT [THb, SO_2_^MSOT^(Air)] and dynamic OE-OT [SO_2_^MSOT^(O_2_), Responding Fraction RF, ΔSO_2_^MSOT^] metrics between the two tumour types. LNCaP tumours showed substantial aggregation of blood near the skin and a significantly higher THb signal than the PC3 (149±12, n_t_=35, n_b_=17 LNCaP vs. 28.7±1.6, n_t_=53, n_b_=30 PC3; p<0.0001), consistent with the haemorrhagic phenotype. The static measurement of SO_2_^MSOT^(Air), commonly used in the literature, suggests both tumours have similar vascular oxygenation on average (Figure 2B; PC3 0.265±0.007, n_t_=53, n_b_=30vs. LNCAP 0.283±0.012, n_t_=35, n_b_=17; p=0.22). Conversely, application of our OE-OT approach reveals highly significant differences between the tumours: the mean SO_2_^MSOT^(O_2_), RF and ΔSO_2_^MSOT^ are all significantly lower for PC3 than LNCaP tumours (Figure 2B; SO_2_^MSOT^(O_2_)=0.308±0.009 vs. 0.370±0.016, p=0.002; RF=0.53±0.02 vs. 0.63±0.05, p=0.04, and ΔSO_2_^MSOT^=0.041±0.005 vs. 0.087±0.011, p=0.0005; n_t_=53, n_b_=30 and n_t_=35, n_b_=17 for PC3 and LNCaP respectively).

**Figure 3:**
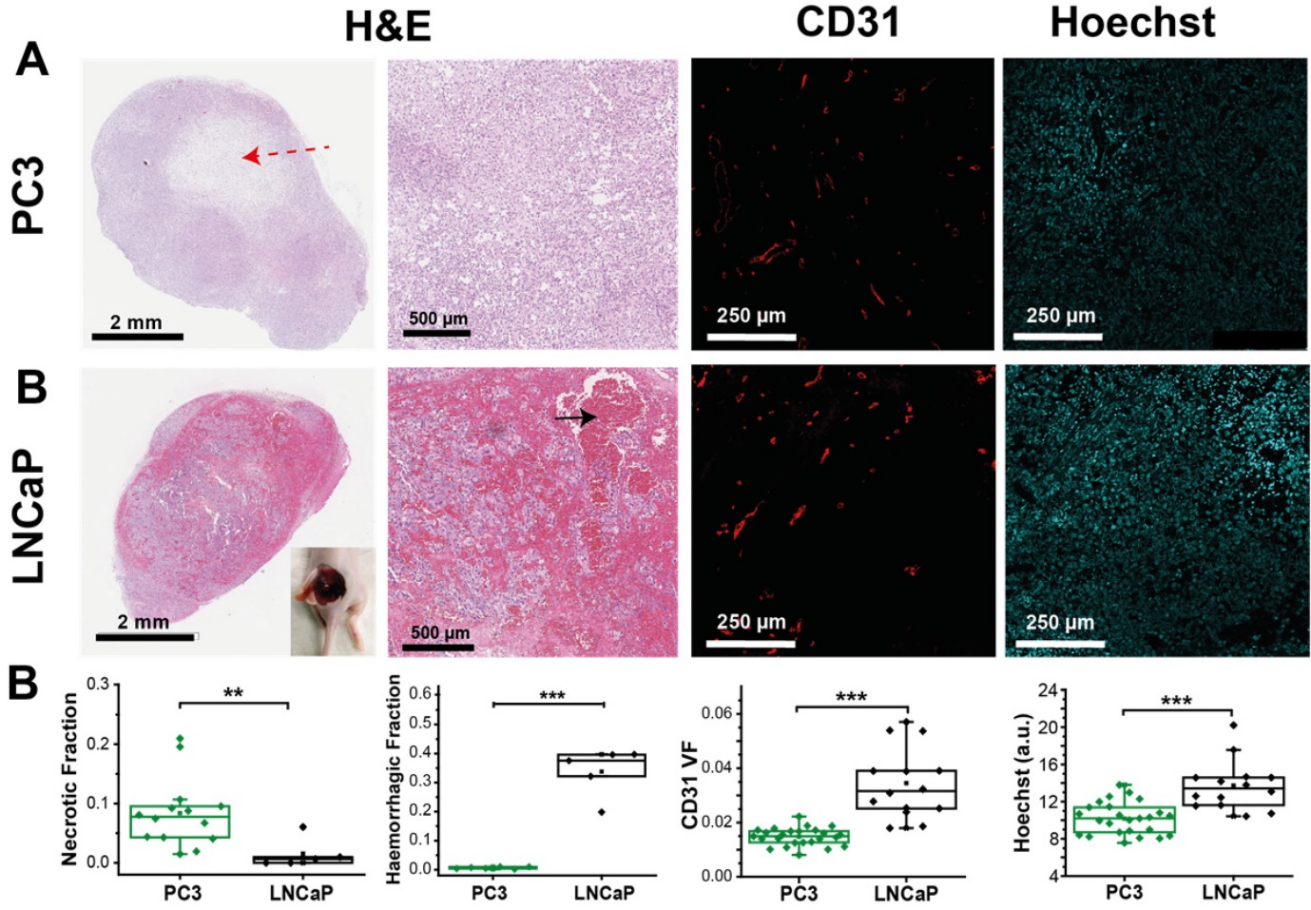
** PC3 and LNCaP tumours show significant differences in morphology, as well as vascular density and function, consistent with OE-OT findings. **(A) Example fields of view from sections stained with H&E, immunofluorescent CD31 and unstained showing Hoechst fluorescence. H&E sections illustrate the characteristic presence of necrotic cores in PC3 tumours (red dashed arrow) and extensive haemorrhage in the LNCaP (black arrow, inset showing haemorrhage in an example LNCaP tumour after removing the skin). Distinct vascular density (CD31) and small molecule agent delivery capabilities (Hoechst) are apparent between the tumour types (n=14,6,25 PC3, n=5,5,25 LNCaP for necrosis, haemorrhage and fluorescence respectively). Each point represents the mean of the given metric over 4 sections for each biological replicate * p<0.05, ** p<0.01, *** p<0.001 by unpaired two-tailed t-test (unequal variances). Box between 25^th^ and 75^th^ percentile, line at median.

**Figure 4:**
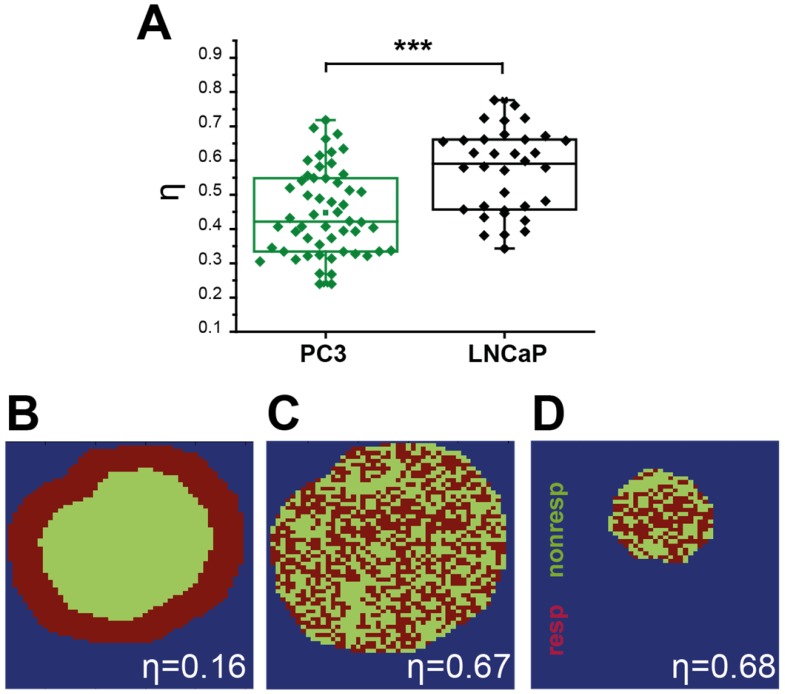
** Quantification of spatial vascular heterogeneity within the tumours. **(A) Box chart comparing the spatial heterogeneity parameter η for PC3 and LNCaP tumours. Example synthetic tumours together with the corresponding η values are shown to illustrate the numerical value of the parameter for a rim-core response to oxygen challenge (B), a scattered response (C), and for a smaller tumour (D) with the same scattered response distribution illustrating the independence from tumour size. * p<0.05, ** p<0.01, *** p<0.001, by unpaired two-tailed t-test (unequal variances), n=53 PC3, n=35 LNCaP. Box between 25^th^ and 75^th^ percentile, line at median.

Interestingly, the spatial distribution of ΔSO_2_^MSOT^ frequently shows high oxygenation change in regions that have relatively low THb across both tumour types. The pixels classed as non-responding (Figure 2A, RF and ΔSO_2_^MSOT^) tend to be present primarily in the core of the PC3 tumours (black arrows, Figure 2A), whereas the LNCaP tumour response is more heterogeneous, with some apparently haemorrhagic areas responding to the challenge (white arrows, Figure 2A) and others remaining unchanged. These findings are consistent with H&E sections, which show homogenous, poorly differentiated PC3 tumours with a necrotic core (Figure 3A), as opposed to the well differentiated, haemorrhagic phenotype with stromal infiltration and viable core in the LNCaP tumours (Figure 3B). Quantification of spatial heterogeneity (see Supplementary Methods) in the tumour responding fraction image is significantly higher in LNCaP tumours (Figure 4A; LNCaP: 0.57±0.02, n_t_=35, n_b_=17, PC3: 0.447±0.017, n_t_=53, n_b_=30; p<0.0001) and the metric of heterogeneity is robust to tumour size differences (Figure 4B-D). In both tumours, the regions that become well oxygenated after the challenge are not simply the areas with more blood in the static THb image. These data indicate that OE-OT reveals a complex dynamic picture of tumour vascular function and heterogeneity not available using static OT alone.

In the context of evaluating disease aggressiveness, we performed a preliminary examination of the few particularly fast and slow growing tumours within each of the PC3 and LNCaP cohorts. ΔSO_2_^MSOT^ derived from the first imaging session (when the tumours had just reached > 30mm^3^) was significantly higher for those tumours that proceeded to grow more rapidly during the remainder of the experiment (PC3: n_b_=3 per group, p<0.001; LNCaP: n_b_=3 per group, p<0.05).

### Tumour response to OE-OT correlates with vessel perfusion and permeability

Having established the robustness of the OE-OT metrics, we then explored their correlation with histopathological assessment of vascular density and function (Figure 3A). PC3 tumours show little CD31 VF or Hoechst uptake on average (Figure 3B) and these parameters are uncorrelated (r=0.25, p=0.21, n=25); conversely, LNCaP tumours show high CD31 VF and Hoechst uptake, which are positively correlated (r=0.63, p=0.02, n=14). Further supporting the low level of vascular functionality in PC3 tumours, CAIX staining (indicative of tumour hypoxia) was over 3-fold higher in these tumours (0.63±0.03 n=6 vs. 0.19±0.03, n=5; p<10^-5^, Figure S8).

Neither static OT nor dynamic OE-OT metrics extracted from the PC3 tumours correlate to CD31 VF (Table 1 left) indicating that not all PC3 tumour blood vessels are functional, as non-perfused vessels will not be seen by OT. SO_2_^MSOT^(O_2_) and ΔSO_2_^MSOT^ show significant negative correlation to tumour necrotic fraction (Figure S9A; n=14; r=-0.67, p=0.008 and r=-0.61, p=0.02 respectively). In PC3 tumours, OE-OT metrics are strongly correlated to Hoechst uptake (Figure S9B), with negative correlations observed for Responding Fraction RF (r=-0.61, p<0.001, n=25), ΔSO_2_^MSOT^ (r=-0.49, p=0.01, n=25) and T_1/2_ (r=0.52, p=0.02, n=20). SO_2_^MSOT^(O_2_) is positively correlated to CD31 Perfused Area (PA) in both tumours as expected (PC3: r=0.45, p=0.03, n=25; LNCaP: r=0.58, p=0.03, n=14). T_1/2_ could not be computed for 4 PC3 tumours due to insufficient change in SO_2_^MSOT^ over the background noise. As expected, these tumours showed a lower Hoechst intensity compared to the rest of the group (11.5±0.9 vs. 10.0±0.3, n=4 vs. n=22, low vs. high responders, p=0.09). LNCaP tumours (Table 1 right) show no significant relationships to tumour necrotic fraction (Figure S10A) or Hoechst uptake (Figure S10B). Unlike PC3 tumours, a positive correlation is observed between CD31 VF and Hb/THb (r=0.54, p=0.046/r=0.50, p=0.07, n=14), indicating that LNCaP vessels are more likely to be blood rich.

**Figure 5:**
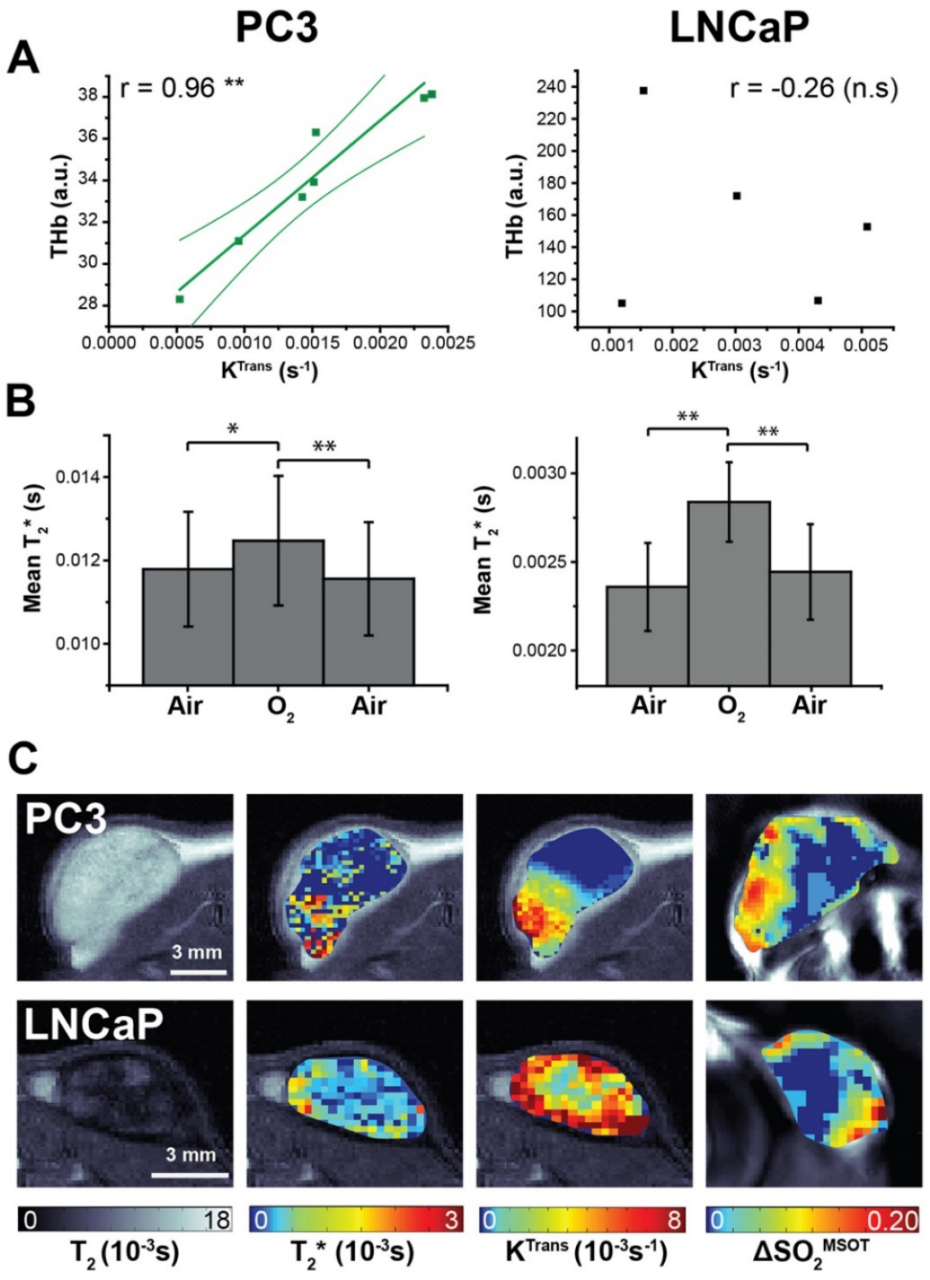
Magnetic resonance imaging indicates differences in K-trans and T_2_* between the tumour types (A) Total haemoglobin, as derived from static OT data, was the only metric to correlate with K-trans, as measured by DCE-MRI. Averaged changes in T_2_* (B) indicate a significant positive response to the oxygen gas challenge as measured by BOLD MRI. Representative images from both tumour types (C) show the spatial distribution patterns in the anatomical T_2_, T_2_*, K^Trans^ and ΔSO_2_^MSOT^ maps. * p<0.05, ** p<0.01, *** p<0.001 by Pearson r correlation (A) or paired two-tailed t-test (B); n=7 PC3, n=5 LNCaP.

DCE- and BOLD-MRI were performed as surrogate markers of perfusion/permeability (via gadolinium uptake quantified with K^Trans^) and oxygenation (via deoxyhaemoglobin T_2_* effects) respectively (Figure 5). K^Trans^ tended to be lower in PC3 tumours than LNCaP, in line with lower Hoechst uptake, although the trend was not significant (0.0014±0.0002s^-1^, n=7 vs. 0.0030±0.0008s^-1^, n=5, p=0.09). THb shows a strong correlation to transfer constant K^Trans 47^ in PC3 tumours (Figure 5A; r=0.95, p<0.003; n=7) but not in LNCaP (r=-0.26, p=0.67; n=5). No correlation with K^Trans^ was observed in any OE-OT metrics, although a monotonic decrease in K^Trans^ was observed with depth in both tumour types (0.0012±0.0003s^-1^ vs. 0.00078±0.00013s^-1^, n=7, p=0.09 PC3 and 0.004±0.0008s^-1^ vs. 0.002±0.0002s^-1^, n=5, p=0.14 LNCaP). The T_2_* in PC3 tumours was significantly longer (0.0095±0.0008s vs. 0.0024±0.0002s, p<10^-5^) indicating T_2_* shortening due to the large haemorrhage in LNCaP tumours. While the mean T_2_* shows significant changes in response to the oxygen challenge in both tumours (Figure 5B; p<0.01 in both cases), no correlation was observed between T_2_* or ΔT_2_* and OT/OE-OT metrics on a per-tumour basis (p>0.18 in both cases). The high morphological heterogeneity of LNCaP tumours is also reflected in their T_2 _maps (Figure 5C).

### OE-OT reveals intra-tumour vascular heterogeneity

**Figure 6:**
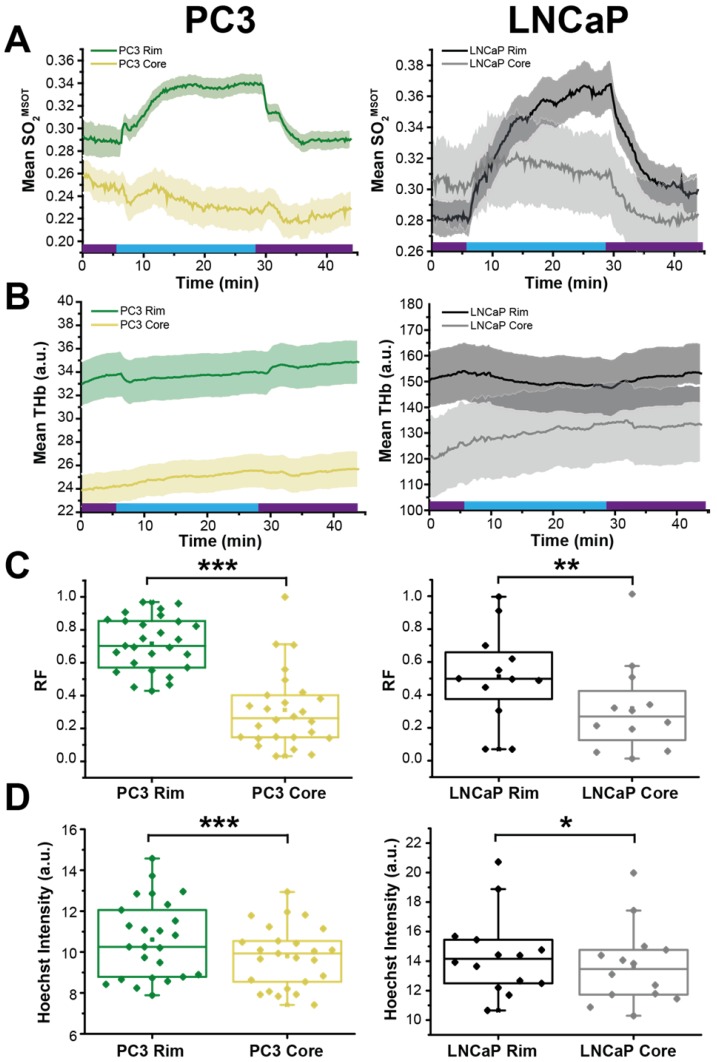
** OE-OT derived hemodynamics highlights tumour rim-core disparity. **SO_2_^MSOT^ (A) and THb (B) in rim and core of PC3 (left) and LNCaP (right) tumours evolves differently, as evidenced by the significant difference in responding fraction (RF, C) and Hoechst Intensity (D). * p<0.05, ** p<0.01, *** p<0.001 by unpaired two-tailed t-test (unequal variances). n=27 PC3, n=12 LNCaP. Box between 25^th^ and 75^th^ percentile, line at median. Air and oxygen breathing schedule in kinetic plots is indicated by purple and blue lines respectively.

Finally, we examined the relationship between OE-OT metrics and vascular dynamics, investigating in detail the rim-core effects observed qualitatively in H&E sections in Figure 3. SO_2_^MSOT^(O_2_), Responding Fraction RF and ΔSO_2_^MSOT^ all decreased significantly with an increase in PC3 tumour size, but were uncoupled from growth in LNCaP tumours (Figure S11; PC3 p<0.003; LNCaP p>0.12). This could not be explained by an increase in necrotic fraction, as there was no relationship in either tumour between size and necrosis (PC3: r=0.19, p=0.49; LNCaP: r=0.74, p=0.15). In an attempt to elucidate the underlying reason for these changes, we compared the OE-OT derived SO_2_^MSOT^ (Figure 6A) and THb (Figure 6B) kinetics in a 1 mm thick tumour rim and the remaining tumour core. These data reveal a dramatic rim-core effect in the PC3 tumours, with little OE-OT response in the core; the LNCaP tumour core responds, albeit with a lower magnitude and different kinetic profile compared to the rim. Both RF (Figure 6C) and Hoechst uptake (Figure 6D) are significantly higher in the rim compared to the core in both tumour types. The maturity of the vasculature, indicated by the ratio of ASMA and CD31 staining, was found to be significantly higher in the 0.5mm rim of PC3 tumours compared to the core (n=6, 0.149±0.010 vs. 0.132±0.011; p=0.03). For LNCaP tumours, no significant difference was seen between rim and core (n=5 sections, 0.181±0.010 vs. 0.201±0.013; p=0.22) but LNCaP tumours showed significantly higher ASMA/CD31 than PC3 tumours (0.184±0.010 vs. 0.132±0.011, p=0.0004).

**Figure 7:**
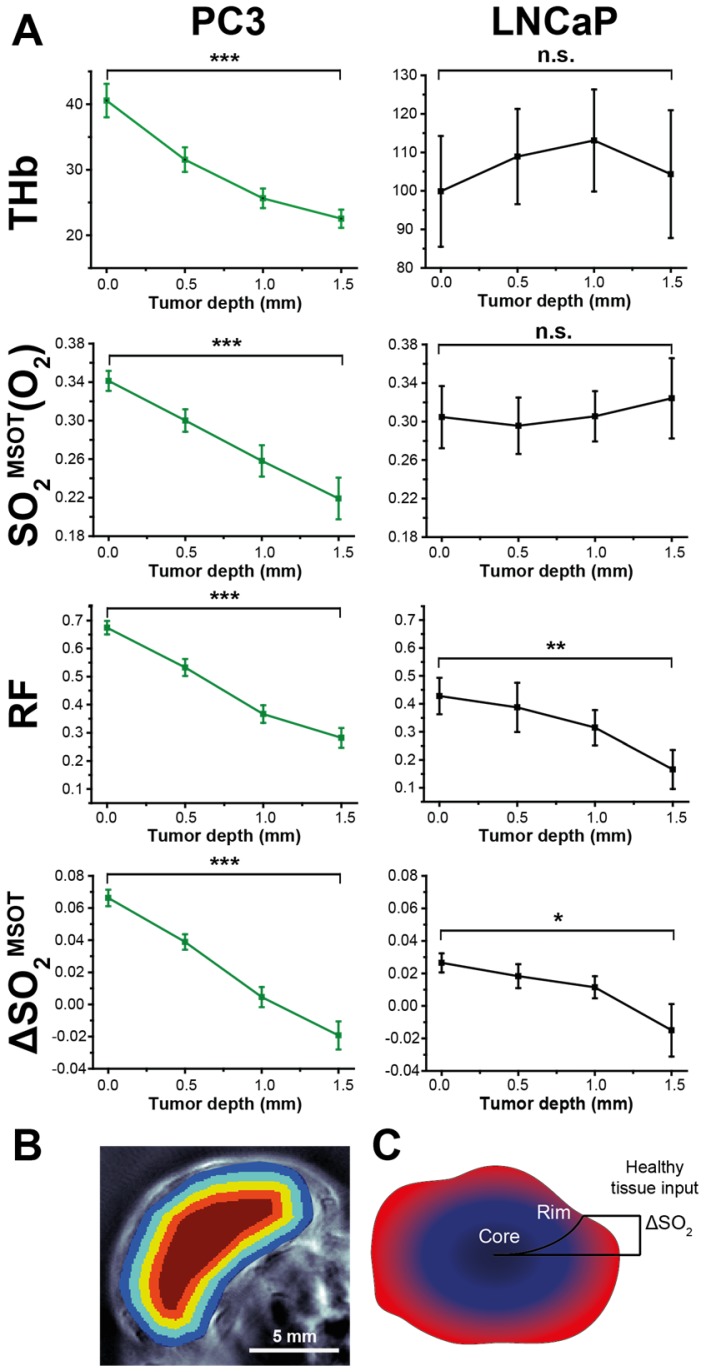
** Depth dependence of static (OT) and dynamic (OE-OT) imaging response. **(A) Mean values across all tumours illustrating the depth dependence of THb and OE-OT metrics, with example segmentation presented in (B) Tumour RF and ΔSO_2_^MSOT^ have significant gradients with depth in both tumour types, as indicated schematically in (C). * p<0.05, ** p<0.01, *** p<0.001 by paired two-tailed t-test between first and last depth measurement; n_t_=34, n_b_=23 PC3, n_t_=7, n_b_=5 LNCaP.

Segmenting the first 2mm of the rim in larger tumours into 0.5 mm concentric regions (see example Figure 7B) reveals a striking pattern: both RF and ΔSO_2_^MSOT ^decrease monotonically with depth in both tumour types, while THb and SO_2_^MSOT^(O_2_) decrease only in PC3 tumours (Figure 7A). *In vitro* measurements indicated that PC3 cells have a significantly higher oxygen consumption than LNCaP cells (1.32±0.08 µs/min vs. 0.77±0.12 µs/min, p=0.006; see Supplementary Methods). Higher oxygen consumption could cause a steeper longitudinal oxygenation gradient in the PC3 tumours, which taken together with the poorer vascular function, further reinforces the rim-core disparity. These OE-OT findings support the differences in vascular function indicated in *ex vivo *analysis, with PC3 tumour vessels being relatively mature and well perfused only in the rim (Figure 7B) while LNCaP tumour vessels are generally functional and perfused in both rim and core.

## Discussion

Optoacoustic tomography (OT) is an emerging modality that reveals haemoglobin concentration and oxygenation with high spatial resolution and sensitivity. OT yields signals even in areas of very low haemoglobin concentration, such as the tumour core, which are often excluded from analysis in non-tomographic studies^29^,^30^,^32^. We hypothesized that by studying the dynamic response of the tumour vasculature to oxygen enhanced (OE)-OT, we would gain greater insight into tumour vascular dynamics and spatial heterogeneity, which could in future be applied in the context of prostate cancer diagnosis and staging^48^, as well as in assisting patient stratification for therapy. OE-OT provides non-invasive imaging of vascular function and heterogeneity, which we validated with *ex vivo* analysis, and shows high repeatability compared to the traditional static OT oxygenation measurements. To establish the potential of OE-OT and define the characteristics that can be measured with the technique, we tested two prostate cancer xenograft models. PC3 tumours have a hypoxic and necrotic core, with relatively low CD31 vessel density, poor ASMA/CD31 coverage and low Hoechst uptake when compared to the rim. Conversely, LNCaP tumours show only a minor rim-core effect, with LNCaP tumours overall exhibiting lower hypoxia and a much higher CD31 vessel density, ASMA/CD31 coverage, Hoechst uptake and K^Trans^ when compared to PC3 tumours. The LNCaP tumours were also highly haemorrhagic, with short T_2_* and large blood lakes visible on H&E.

While static OT measurement of total haemoglobin revealed a higher blood content in LNCaP tumours, the static oxygenation metric SO_2_^MSOT^(Air), frequently used in the literature^29^,^32^,^49^ was identical in the two tumour types, apparently insensitive to the striking differences in their vasculature. Importantly, OE-OT overcame this limitation and revealed highly significant differences between the two xenografts, complementing previous MRI studies that demonstrated the potential of an oxygen challenge to reveal additional insight into tumour haemodynamics and enhance contrast between models with distinct vascularity^13^,^50^ During OE-OT, the SO_2_^MSOT^ signal in LNCaP tumours reaches a high plateau, reflecting the overall higher total haemoglobin concentration and lower oxygen consumption measured in the LNCaP than in PC3 cells. Only a mild rim-core effect was observed, evidenced in OE-OT metrics *in vivo *and Hoechst uptake *ex vivo*. The histopathological heterogeneity both within and between LNCaP tumours was revealed by a spatially heterogeneous response to the oxygen challenge *in vivo* (even in some regions of haemorrhage) with both OE-OT and MRI. Taken together, these data indicate that the OE-OT response of LNCaP tumours was consistent with a generally well perfused, viable tumour containing marked areas of haemorrhage.

PC3 tumours exhibit a pronounced rim-core effect^32^,^51^ with little or no OE-OT response occurring in the hypoxic and necrotic core. OE-OT in the rim, however, reveals a rapid rise in SO_2_^MSOT^ following the oxygen challenge, expected given the presence of higher ASMA/CD31 and K^Trans^ in the rim vasculature. In functional vasculature, the influx of oxygenated blood results in vasoconstriction^52^, likely manifested in the initial SO_2_^MSOT^ jump and associated drop in THb signal, due to a decrease in vascular area. Taken together, these data indicate that the OE-OT response of PC3 tumours was consistent with a hypoxic and necrotic core, but a mature and perfused rim vasculature. PC3s also show per-tumour correlations of OE-OT (but not OT) metrics to histopathological analysis, highlighting the added value of OE-OT. SO_2_^MSOT^(O_2_) shows a strong inverse relationship to the tumour necrotic fraction. Responding Fraction RF and T_1/2 _show a strong inverse relationship to Hoechst uptake. ΔSO_2_^MSOT^ is modulated by both necrosis and Hoechst uptake. These histological correlations were not seen in the LNCaP tumours, most likely due to their haemorrhagic phenotype, which enforces a more diffusion-based oxygen delivery mechanism and marginalises the effect of vascular function. The LNCaP tumours appear to rely instead on high vascularisation and perfusion throughout the tumour mass; the Hoechst uptake in LNCaP was positively correlated to CD31 vessel density, indicating a largely perfused vascular network. To the best of our knowledge, this is the first study to perform biological validation of optoacoustic metrics by using correlations on a per-tumour basis.

Spatial and temporal heterogeneity in the vasculature of clinical and experimental tumours is high^2^ but is considered to have substantial prognostic value^53^. In this regard, the spatial resolution of OE-OT provides unique insight into vascular heterogeneity. The hypoxic PC3 tumour core^4^ has been shown previously to exhibit reduced vascular volume and high permeability due to the secretion of angiogenic growth factors^51^. This would result in a steep oxygenation gradient in the tumour vasculature from rim to core, as observed. Hoechst uptake, used here to assess vessel function and providing a general indication of delivery of small molecule agents such as therapeutics, is a mixed measure of vascular perfusion and permeability; in the core it is most likely a reflection of high permeability of relatively immature core vasculature^2^, as confirmed by a low ASMA/CD31 coverage, resulting in a slow or negligible OE-OT response. This likely drives the inverse relationship between tumour Responding Fraction RF and Hoechst uptake. The PC3 tumour rims are likely to have high Hoechst uptake more due to high perfusion^4^, resulting from mature rim vasculature. The inverse relationship between T_1/2_ and Hoechst uptake is clearly explained in this case, since tumour tissue with a short T_1/2 _is more similar to healthy tissue vasculature and hence should be well perfused. This vascular phenotype in the rim may also explain the negative ΔSO_2_^MSOT^ measured in the core, as vasoconstriction and rapid oxygen consumption in the rim^7^ would result in decreased oxygen delivery in the core. OE-OT therefore reveals a complex picture of tumour vascular dynamics.

The ability of OE-OT to reveal heterogeneity in vascular density and function during tumour development has broad clinical implications. A key unmet need in early cancer detection, especially in the prostate, is delineating those tumours that will progress to invasive disease from those that will remain indolent. In a preliminary analysis, we found that the magnitude of the response to the gas challenge was significantly higher in the first imaging session for those tumours that proceeded to grow rapidly (in both PC3 and LNCaP). If confirmed in a larger cohort of mice, given the prognostic significance of vascular heterogeneity and tumour hypoxia, OE-OT may potentially be applied in early disease to reduce overtreatment. Furthermore, the correlation between OE-OT metrics in PC3 tumours with Hoechst uptake suggests that OE-OT may have value in predicting delivery of small molecule therapeutic agents in specific tumour types, improving patient stratification for chemotherapy.

We have shown that dynamic OE-OT metrics exhibit high repeatability, clearly outperforming the static metrics and providing a high degree of confidence in the precision of the measurements, which is crucial for clinical translation. Nonetheless, there remain some limitations that must be addressed in future studies. Tumour response to an oxygen challenge is a combination of several physiological phenomena, both systemic and local to the tumour. The observed oxygen saturation is influenced systemically by applied anaesthesia and locally by transport and consumption of oxygen in the tumour, depending on factors such as blood flow and pH^54^. Taking OE-OT forward into clinical applications will remove the need for anaesthesia, but the influence of local factors will need to be understood. The method should be further validated in a more realistic transgenic tumour model, as the vascularisation of subcutaneous models differs from that of spontaneous tumours^55^. OE-OT metrics did not correlate with DCE- or BOLD-MRI when comparing mean values per-tumour. Spatial co-registration of the OE-OT and MRI data in a larger cohort may therefore be necessary to elucidate such relationships. To test the performance of OE-OT we chose to examine two tumour models with clear differences in aggressiveness and growth rates; as a result of the disparity in tumour volumes, size matching was not performed. Instead, the size dependence was examined and accounted for in our analysis. Our OE-OT data is not corrected for the effects of fluence; in the haemorrhagic LNCaP model spectral colouring may impact the absolute quantification of SO_2_. Methods to perform fluence correction of OT data have received only limited validation *in vivo*^56^; future work should aim to directly relate OT data to absorbed optical energy density and hence enable a direct readout of absolute SO_2_.

There remains an unmet clinical need for visualisation and robust quantification of tumour vascular function in the context of assessing prognosis and therapeutic response in solid tumours. Significant advances have been made towards clinical application of optoacoustic imaging^57^. In prostate cancer, early results indicate that OT may be useful to distinguish malignant from benign disease^58^,^48^; development of transrectal clinical probes is underway^59^,^60^. Capitalizing on these recent advances in optoacoustic tomography, we have developed a non-invasive and clinically translatable technique to assess tumour vascular dynamics and heterogeneity without the need for intravenous injection of a contrast agent. Our OE-OT metrics can distinguish between PC3 and LNCaP tumours based on their vascular characteristics. OE-OT metrics showed high repeatability and correlated directly to tumour vessel function assessed *ex vivo, *directly relevant for prostate cancer staging and therapeutic monitoring, which has not been shown before in an optoacoustic study. Importantly, OE-OT reveals a complex spatiotemporal picture of tumour vascular function, enabling us to extract haemoglobin concentration and oxygenation from static OT, along with data related to vasoactivity, perfusion and permeability in OE-OT. OE-OT has been applied here in the preclinical setting, but with future hardware developments could be integrated into existing transrectal ultrasound probes for prostate cancer^59^,^60^, to improve non-invasive delineation of aggressive and indolent disease and assist with patient stratification for therapy^48^,^61^.

## Figures and Tables

**Table 1:**
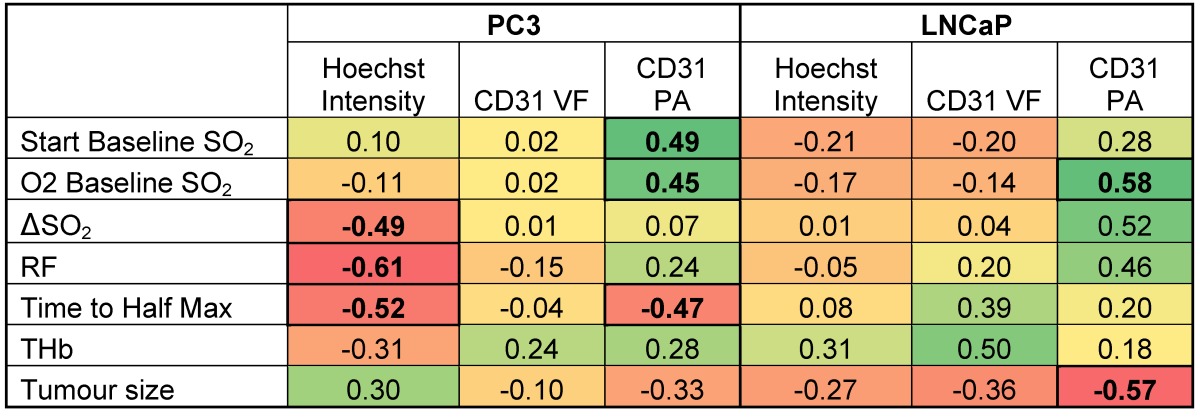
Correlations of OT and OE-OT metrics to histology for PC3 and LNCaP tumours.

Pearson r correlation coefficients are shown with relationships significant at a p<0.05 level highlighted in bold. Color-coding is designed to illustrate the magnitude and direction of the correlations (positive correlation = green, negative correlation = red).
