# Supplementary material for: Oxygen Enhanced Optoacoustic Tomography (OE-OT) Reveals Vascular Dynamics in Murine Models of Prostate Cancer
Source: Theranostics. 2017 Jul 8;7(11):2900–13. doi: 10.7150/thno.19841 (PMC5562224; doi:10.7150/thno.19841)

Supplementary Information for: Oxygen enhanced optoacoustic tomography (OE-OT) reveals vascular dynamics in murine models of prostate cancer

Michal R Tomaszewski[1,2], Isabel Quiros Gonzalez[1,2], James O'Connor[3,4], Oshaani Abeyakoon[5], Geoff JM Parker[6,7], Kaye Williams[8], Fiona Gilbert[5] and Sarah E Bohndiek[1,2]*

[1] Department of Physics, University of Cambridge, U.K.

[2] Cancer Research UK Cambridge Institute, University of Cambridge, U.K.

[3] Institute of Cancer Sciences, University of Manchester, UK.

[4] Department of Radiology, The Christie NHS Foundation Trust, UK.

[5] Department of Radiology, University of Cambridge, U.K.

[6] Centre for Imaging Sciences, University of Manchester, U.K.

[7] Bioxydyn Limited, Manchester, UK

[8] Manchester Pharmacy School, University of Manchester, UK.

Supplementary Methods

BOLD and DCE MRI data acquisition

BOLD data were acquired using a spoiled multiple gradient-echo sequence (FOV 40mm, 2mm slice, 128x128 points, TR 200ms, time to first echo 2.5ms, echo spacing 2.5ms, 8 averages, 8 echoes, excitation pulse 45° sinc of duration 1ms). Both RF spoiling (phase increment 117°) and gradient spoiling (11.5G/cm, duration 0.356ms) were employed. BOLD data were captured directly before each gas switch. T_2* values were calculated using the scanner software.

DCE-MRI data were acquired using a spoiled gradient echo sequence (FOV 40mm, 2mm slice, 128x128 points, TR 20ms, TE 1.62ms, 2 averages, excitation pulse 20° gauss of duration 0.8ms). As before, both RF spoiling (phase increment 117°) and gradient spoiling (7.68G/cm, duration 0.5ms) were employed. 10 images were acquired during the 1 minute prior to administration of contrast agent to provide a baseline reference and 120 images were acquired in the 11 minutes after injection. Baseline T_1 data were acquired using non-selective adiabatic inversion recovery followed by a centric phase-encoding single-shot turbo FLASH acquisition (FOV 40mm, 2mm slice, 128x128 points, TR 4ms, TE 1.71ms, 4 averages, excitation pulse 5° gauss of duration 1ms, inversion times 0.05, 0.1, 0.2, 0.5, 1.0, 2.0, 5.0 and 8.0 seconds, with a 12 second relaxation delay between images).

Oxylite Measurements

Mice were anesthetized, placed on a heat pad and prepared breathing medical air. Care was taken to maintain the temperature of the animal at ~37°C. The tumour and hind leg area were immobilized using surgical tape, and while the tumour was held with tweezers, a 23G needle was inserted 2-3mm under the skin. The needle was then retracted and replaced with a pO_2 and flow Oxylite probe, which was subsequently also fixed in place to minimize movement. A similar procedure was repeated on a muscular area of the hind leg, where a pO_2 and temperature probe was inserted. A time resolved measurement was then initiated, and

after baseline stabilization, the respiratory gas was switched to pure oxygen as per the optoacoustic imaging experiment. If a response was seen, approximately 20 minutes later the respiratory gas was switched back to air.

Histopathologic and immunofluorescent image analysis

Necrotic regions in H&E images were identified with regions of interest drawn by hand in Imagescope (Aperio Technologies Ltd). The necrotic fraction was quantified as the ratio of total necrotic area to total tumour area across a whole section and 4 sections per tumour were analysed. ASMA coverage of the areas marked as CD31 positive using immunohistochemistry in directly adjacent sections was quantified using the Halo (Indica Labs) image analysis platform. The outermost 0.5mm rim area was excluded due to the presence of healthy tissue and skin around the tumour. Sections were then automatically divided into 3 regions: two concentric rim areas of 0.5 mm thickness each, and the remaining core. The ASMA on CD31 area divided by total CD31 area (ASMA/CD31) was calculated to given an indication of the vessel coverage of smooth muscle. Cellular regions stained positive for CAIX were identified in each section, again using Halo platform, and the total tissue section area was calculated. The CAIX area was then divided by the total tissue section area to give an indication of the hypoxic area fraction.

Hoechst uptake and CD31 staining were analysed using 4 sections per tumour and up to 10 imaging fields of view per section. Data was considered as an average over the whole tumour section, as well as divided between the rim (fields of view taken at most 1mm from the surface) and core (all remaining fields of view) for subsequent analysis. Identification of tumour blood vessels was performed in the following way: after denoising with 2 pixel 2D median filter and performing a background subtraction using a 30 pixel 2D median filter, CD31 images were thresholded using an automated Otsu thresholding method (‘graythresh’ function in MATLAB 2014a)[1]. Positive areas smaller than 25 μm^2 or larger than 12000 μm^2 were filtered out; the remaining areas were expanded to ensure that vessel ellipsoids closed and the holes were filled.

Mice excluded from statistical analysis.

Out of the 33 PC3 tumours implanted, 33 grew and 3 were excluded. Out of 30 LNCaP tumours implanted, 19 grew and 4 were excluded. Data were only excluded from the study analysis if one of the following events occurred: adverse reaction to anaesthesia, showing as persistent decrease in respiration rate or a strong overall downward trend in the SO_2; failed intravenous contrast injection; incorrect sample processing for histology, including problems with sectioning, staining or storage of the samples. If a technical problem was encountered, the OT scan was not analysed, and where possible the measurement was repeated. Data excluded from the final analysis presented are indicated in Table S1. For the calculation of $T_{1/2}$ 5 more data points were excluded (all from PC3 cohort), as the maximum signal was not significantly different from the baseline. This resulted in 4 data points being excluded from $T_{1/2}$ repeatability analysis, indicating poor robustness of the metric.

Measurement of oxygen consumption in cell lines.

Oxygen consumption of the PC3 and LNCaP cells was assessed by the *MitoXpress Xtra Oxygen Consumption (HS Method)* assay (LuxCel Bioscience). Cells were seeded into a 96-well plate in 2 concentrations (80,000 and 90,000 cells/well for PC3 and 70,000 and 80,000 cells/well for LNCaP), with 15 replicates per cell number, in 150 µL of complete media, and incubated overnight. The next day the media was changed, and 10 µL of the MitoXpress Xtra vial reconstituted in 1mL of fresh complete media was added to each well, except 6 blank control wells with no cells that contained media only. Two drops (~150 µL) of pre-warmed HS Mineral Oil were added to each well to prevent oxygen exchange with the surroundings. 3 of the wells with no cells were used as an overall positive control with 1 mg/mL of Glucose Oxidase addition. For each cell line and concentration, 1 µL of 150 µM Antimycin A (Sigma Aldrich) solution was added to 3 wells serve as a cell negative control, and 5 µL of 150 µM Carbonyl cyanide-4-(trifluoromethoxy)phenylhydrazone (FCCP) (Sigma Aldrich) solution was added to another 3 wells for cell positive control. The plate was kept on a heated block throughout the procedure to ensure stable temperature of $37^{O}C$. Afterwards, the plate was

transferred into a plate reader (CLARIOstar, BMG Labtech), where it was scanned for 2.5 hours according to a protocol described elsewhere [2]. The slopes of fluorescent lifetime changes were extracted for each cell line and concentration were extracted as a quantification of the oxygen consumption in the well.

A duplicate plate was used to quantify the relative cell densities in the wells. The plate was incubated for the same time as the plate used for the oxygen measurements. Then the media was aspirated and the plate was frozen at -80°C for 3h and thawed. 50μL of TNE buffer with 20 μg/mL Hoechst 33342 was added to each well, and the plate was incubated at room temperature for 15 minutes. Afterwards the plate was imaged under a plate reader (CLARIOstar, BMG Labtech) at 346nm and 477nm, and the ratio of the fluorescence from the two channels (after blank correction) was used as a measure of the cell density.

Cell counting based on Hoechst fluorescence showed PC3 to have lower densities than LNCaP [(38±8)$x10^3$ and (45±10)$x10^3$ fluorescent arbitrary units for each cell count in PC3 and (55±12)$x10^3$ and 56±12 $x10^3$ fluorescent arbitrary units for each cell count in LNCaP]. The MitoXpress assay showed a significantly higher oxygen consumption in the PC3 wells than in the LNCaP wells (1.32±0.08 and 1.37±0.12 μs/min for PC3 and 0.77±0.12 and 0.94±0.06 μs/min for LNCaP, p=0.002 and p=0.006 respectively).

Quantification of tumour spatial heterogeneity.

The spatial distribution of the OE-OT response was qualitatively different, with the PC3 tumours divided into larger regions of responding pixels including a strong rim-core effect, while for LNCaP tumours, the response was more scattered and heterogeneous. In order to quantify this difference, we defined the metric η as the sum of the perimeters of all the responding regions normalized by the sum of their areas. η was calculated for each tumour RF image using custom software written in MATLAB 2014b. Tumours showing a scattered pattern of small responding regions yield a higher η value than large concentrated regions of response. The metric was tested on a synthetic dataset including a tumour where response

was concentrated in the rim (Figure S7B) and another of the same size with the same overall RF but with the response scattered randomly (Figure S7C, generated using a MATLAB ‘rand’). η for the first tumour (η=0.16) was indeed much lower than for the second (η=0.67). An arbitrary small subsection of the scattered response tumour was also tested (Figure S7D) to look at the influence of tumour size; η=0.68 indicated that the metric is unlikely to be affected by scaling due to tumour growth.

1. Otsu N. A Threshold Selection Method from Gray-Level Histograms. IEEE Trans. Syst. Man. Cybern. 1979;9:62–6.
2. Hynes J, Nadanaciva S, Swiss R et al. A high-throughput dual parameter assay for assessing drug-induced mitochondrial dysfunction provides additional predictivity over two established mitochondrial toxicity assays. Toxicol. Vitr. 2013;27:560–9.

Supplementary Table

Table S1:

Mouse #	Mouse ID	# Tumours (# Scans)	Cell line	Reason for exclusion
1	3821 2L	1 (2)	PC3	Repeated adverse reaction to anaesthesia
2	0767 1L	1 (1)	PC3	Histology-fixation and sectioning issue
3	0766 1B	1 (1)	PC3	Mouse visibly sick due to tumour burden, adverse reaction to anaesthesia
4	0768 NM	1 (1)	PC3	Large ulcer and scar on the tumour
5	0768 2L	1 (1)	PC3	Problem with the freezing and sectioning of the tumour
6	6705 2L	1 (1)	PC3	Mouse visibly sick due to tumour burden, adverse reaction to anaesthesia
7	6704 NM	1 (1)	PC3	No contrast agent injection, unable to cannulate
8	3085 1L	1 (1)	LNCAP	No contrast agent injection, unable to cannulate
9	6460 2L	2 (4)	LNCAP	Mouse sick, large haemorrhage from the tumour spilling over, adverse reaction to anaesthesia
10	3084 1L	1 (1)	LNCAP	Incorrect storage, sample thawed, unable to section

Supplementary Figures

Figure S1: Tumour growth curves of PC3 (A) and LNCaP (B) tumours in BALB/c nude mice as measured by callipers. Doubling time was 10.9±1.4 days for PC3 and 19±2 days for LNCaP.

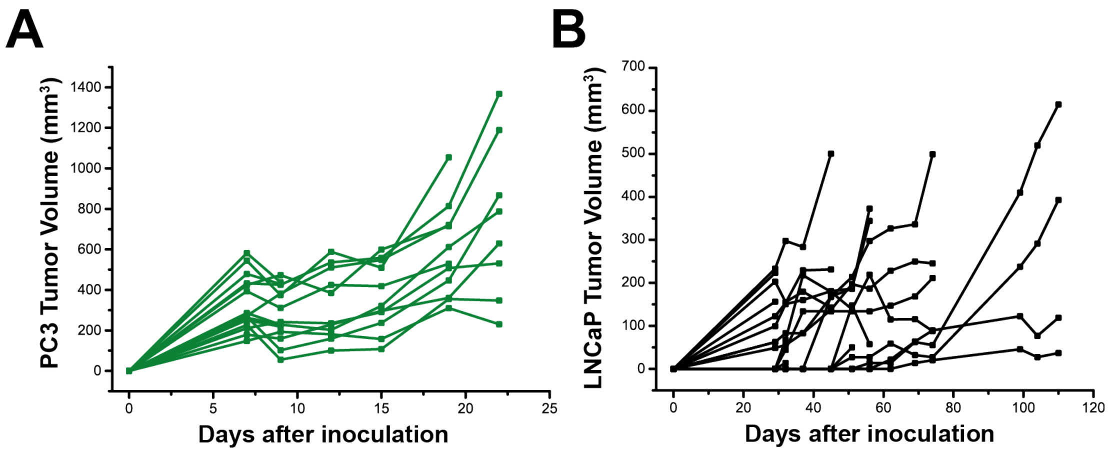

Figure S2: Image of raw optoacoustic tomography metrics oxy- (HbO_2) and deoxy-haemoglobin (Hb) in an air breathing mouse. Example images of a transverse slice through entire mouse bearing a PC3 (left) and LNCaP (right) tumours. The images shown are the raw spectral unmixing outputs of HbO_2 (top) or Hb (middle), and the grey scale absorption map at 800nm ilumination used for Region of Interest (ROI) drawing (bottom). The tumour ROI is outlined in white and healthy tissue ROI used for comparison around the spine is outlined in green. Z axis scales are in arbitrary units.

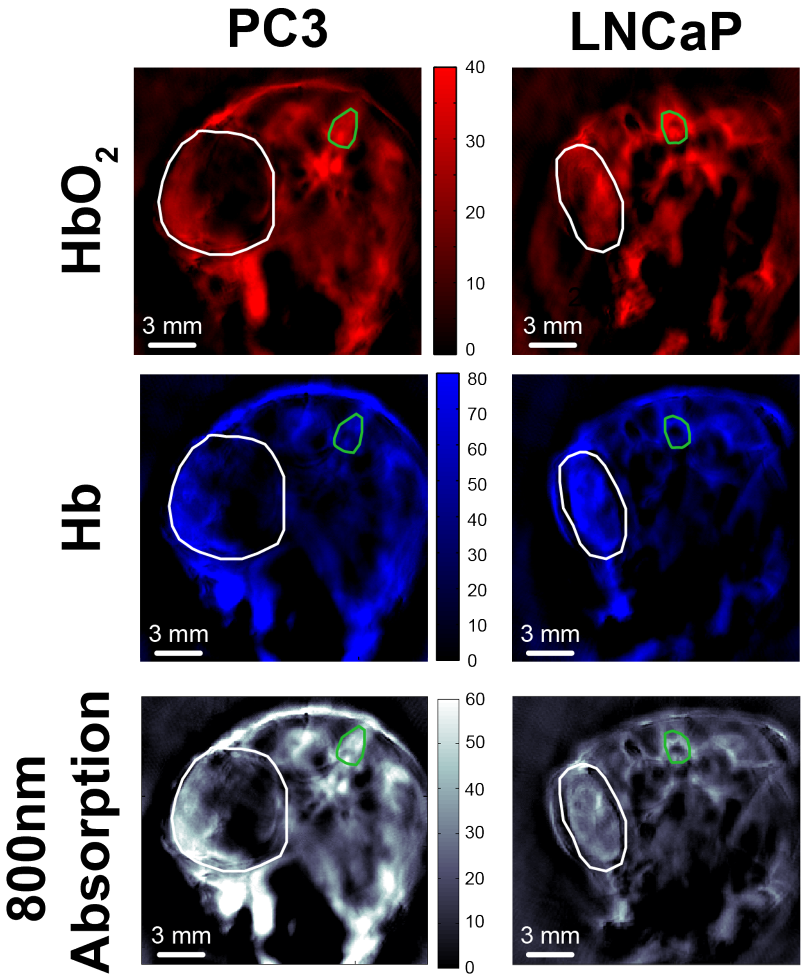

Figure S3: Responding Fraction (RF) quantification is not influenced by threshold choice. Scatter plot showing the RF values calculated using response thresholds of 0 (y-axis) and 0.03 (x-axis), display a clear linear relationship, showing that the quantification is not strongly affected by the change of threshold value, even if an extreme value of 0 is chosen. *** p<0.001 by Pearson rank test. n=8 PC3. Line of best fit and 95% confidence intervals displayed.

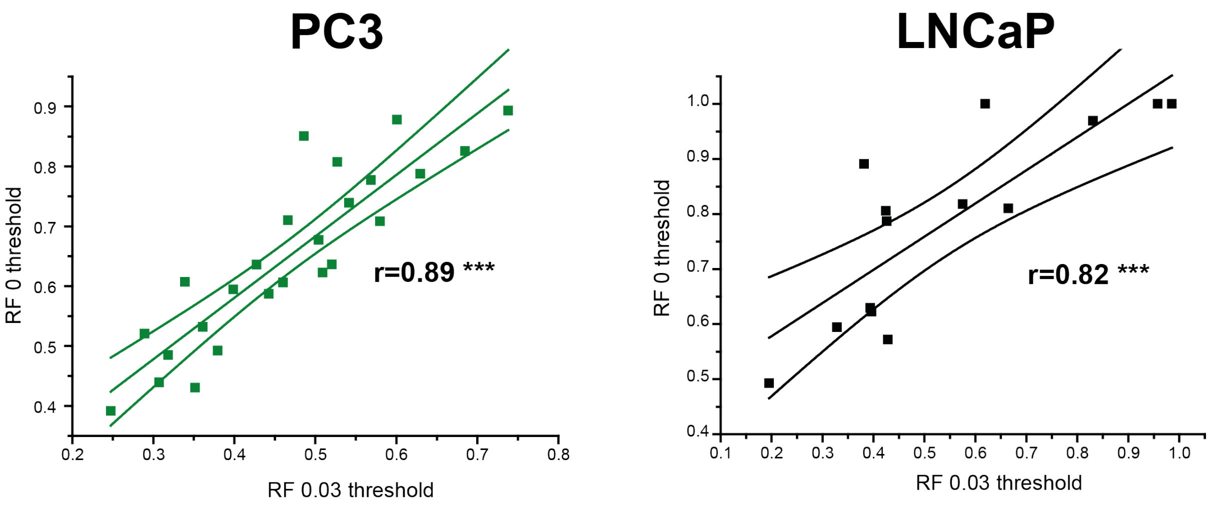

Figure S4: CD31 image analysis algorithm. Unprocessed images of CD31 immunofluorescent sections (left) were denoised and thresholded using an automated Otsu method (centre). Positive areas between 25 and 12000 μm^2 were expanded to ensure that vessel ellipsoids closed and holes were filled (right) and the total vessel area to image area was quantified as 'CD31 Vascular Area Fraction' (CD31 VF).

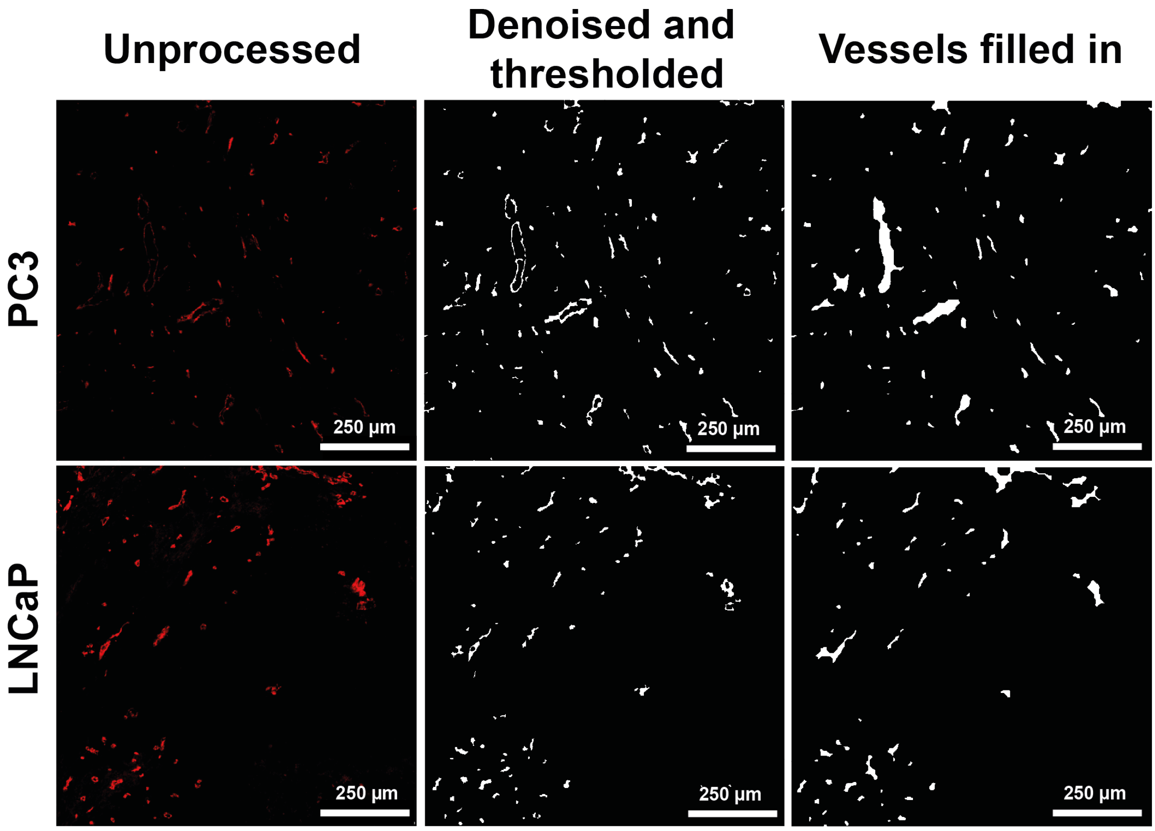

Figure S5: Oxylite pO_2 measurements. (A) pO_2 measured in two representative PC3 tumours subjected to an oxygen gas challenge. (B) pO_2 measured in healthy muscle tissue in two mice subjected to an oxygen gas challenge. Red dotted line indicates onset of the oxygen gas challenge. The second dotted line, colour coded for each mouse, indicates the return to breathing medical air.

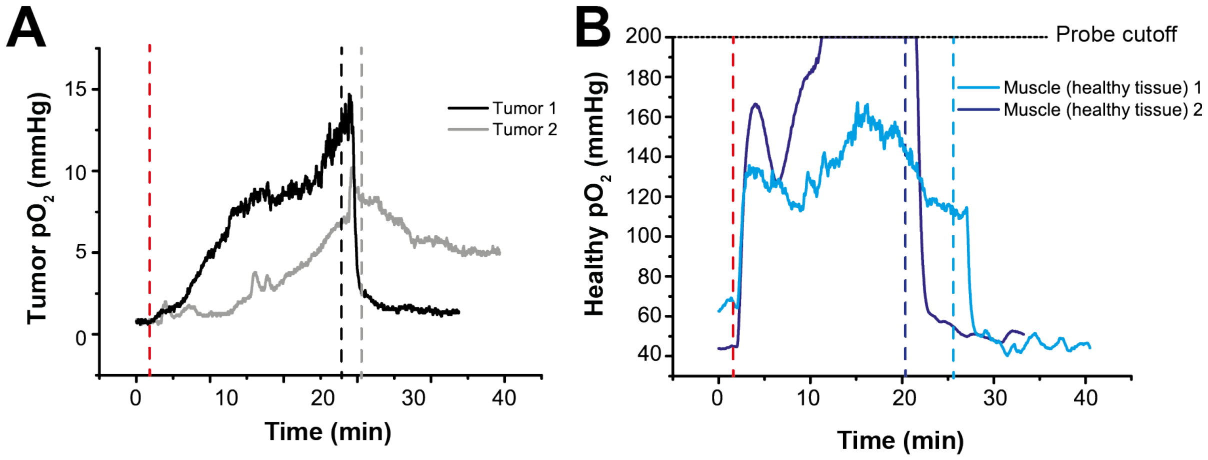

Figure S6: Time evolution of the raw optoacoustic tomography metrics. Kinetics of raw oxyhaemoglobin (A, HbO_2) and deoxyhaemoglobin (B, Hb) signals trend as expected, with Hb showing the inverse trend to HbO_2. (C) Kinetics of the total haemoglobin (THb) signal in the tumour. (D) Kinetics of the THb signal from the healthy tissue region. In the absence of an oxygen gas challenge, SO_2^{MSOT} (E) and THb (F) are constant. Standard error of the mean indicated by the shaded envelope (n_t=53, n_b=30 PC3, n_t=35, n_b=17 LNCaP). Air and oxygen breathing schedule is indicated by purple and blue lines respectively.

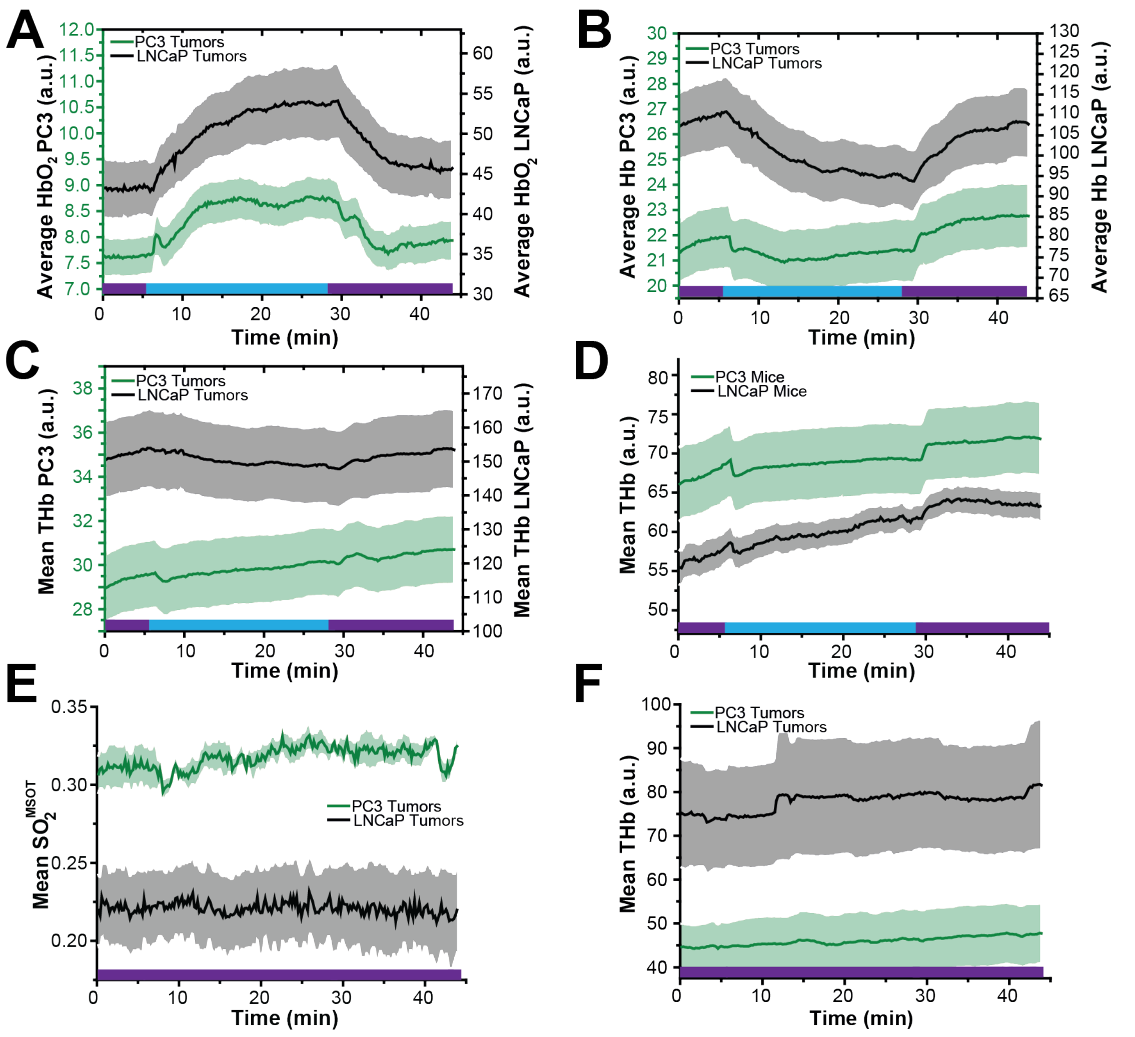

Figure S7: OE-OT metrics show high test-retest repeatability compared to static OT. Each metric was assessed by performing repeated scans on a subset of PC3 tumours 24 hours after the initial oxygen gas challenge. (A) Test-retest values and (B) correlation between repeated measurements. The Pearson correlation coefficients and significance levels are indicated on graphs in (B). * $p<0.05$, ** $p<0.01$, *** $p<0.001$ by Pearson rank test. n=8 PC3. Line of best fit and 95% confidence intervals displayed.

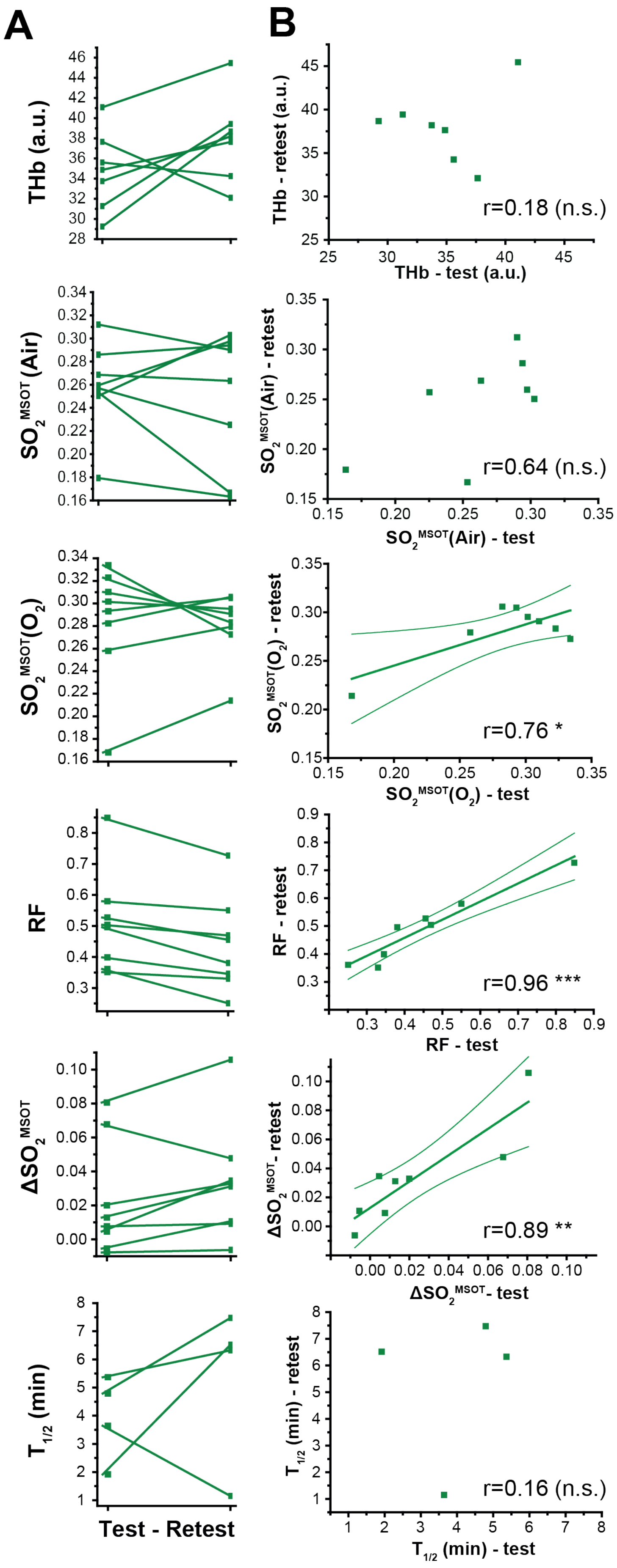
A
B
THb (a.u.)
THb - retest (a.u.)
THb - test (a.u.)
r=0.18 (n.s.)
SO2MSOT(Air)
SO2MSOT(Air) - retest
SO2MSOT(Air) - test
r=0.64 (n.s.)
SO2MSOT(O2)
SO2MSOT(O2) - retest
SO2MSOT(O2) - test
r=0.76 *
RF
RF - retest
RF - test
r=0.96 ***
ΔSO2MSOT
ΔSO2MSOT - retest
ΔSO2MSOT - test
r=0.89 **
T1/2 (min)
T1/2 (min) - retest
T1/2 (min) - test
r=0.16 (n.s.)
Test - Retest

Figure S8: CAIX staining suggest higher hypoxia in PC3 tumours. Representative images of CAIX stained PC3 and LNCaP sections (A), show a marked difference between the cell lines, confirmed in the box plot (B). *** $p<0.001$ by unpaired two-tailed t-test (unequal variances), n=7 PC3, n=5 LNCaP. Box between 25th and 75th percentile, line at median.

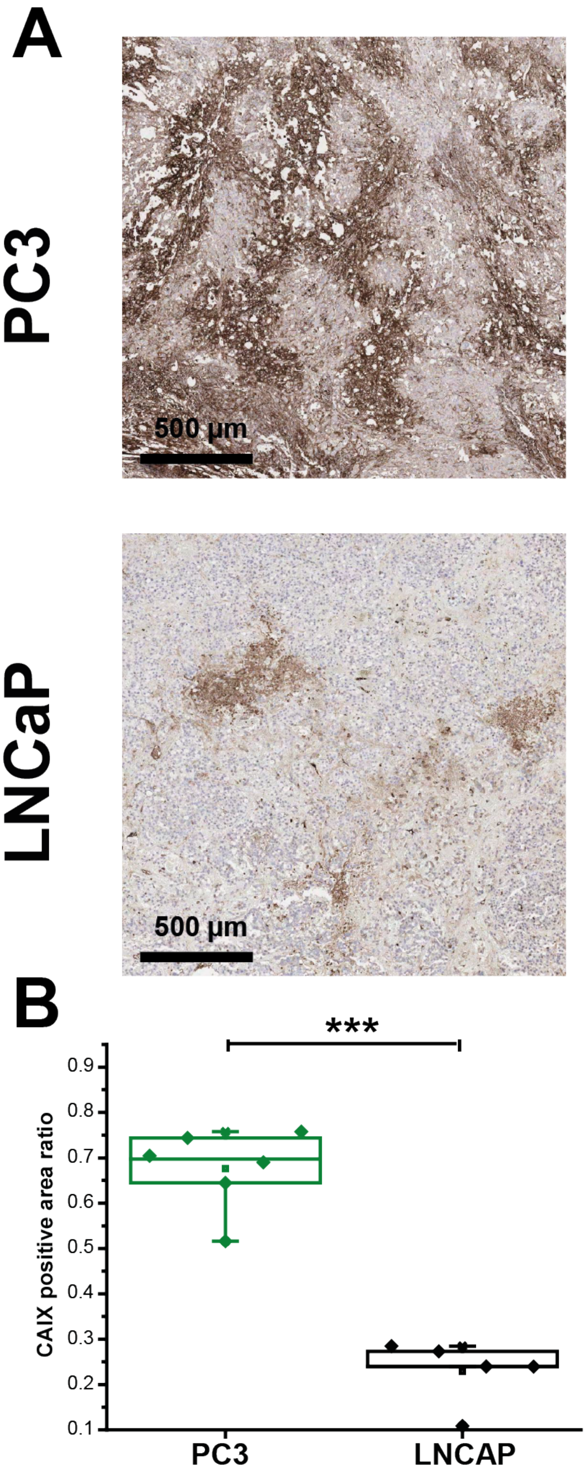

Figure S9: Correlations of metrics derived from PC3 tumours to histopathology. Correlations were tested on a per-tumour basis for relationship to tumour necrotic fraction (A) and Hoechst Intensity (B). The Pearson correlation coefficients and significance levels are indicated on the graphs as * p<0.05, ** p<0.01, *** p<0.001. Line of best fit and 95% confidence intervals displayed where appropriate.

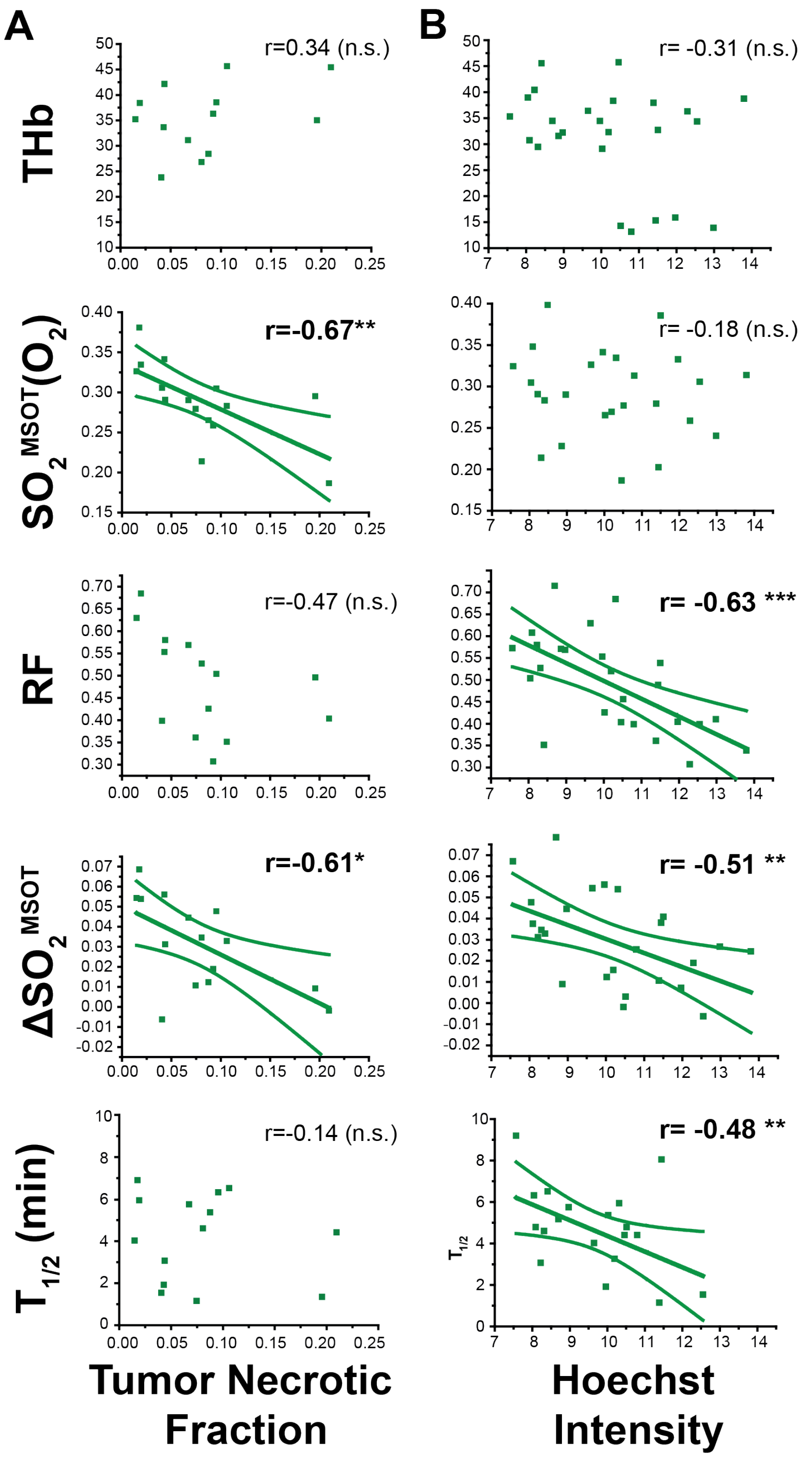

Figure S10: Correlations of mean OT metrics derived from LNCaP tumours to histopathology. Correlations were tested on a per-tumour basis for relationship to tumour necrotic fraction (A) and Hoechst Intensity (B). The Pearson correlation coefficients and significance levels are indicated on the graphs. Due to poor robustness of $T_{1/2}$ metric in larger LNCaP tumours only 2 points can be shown for the necrotic fraction relationship.

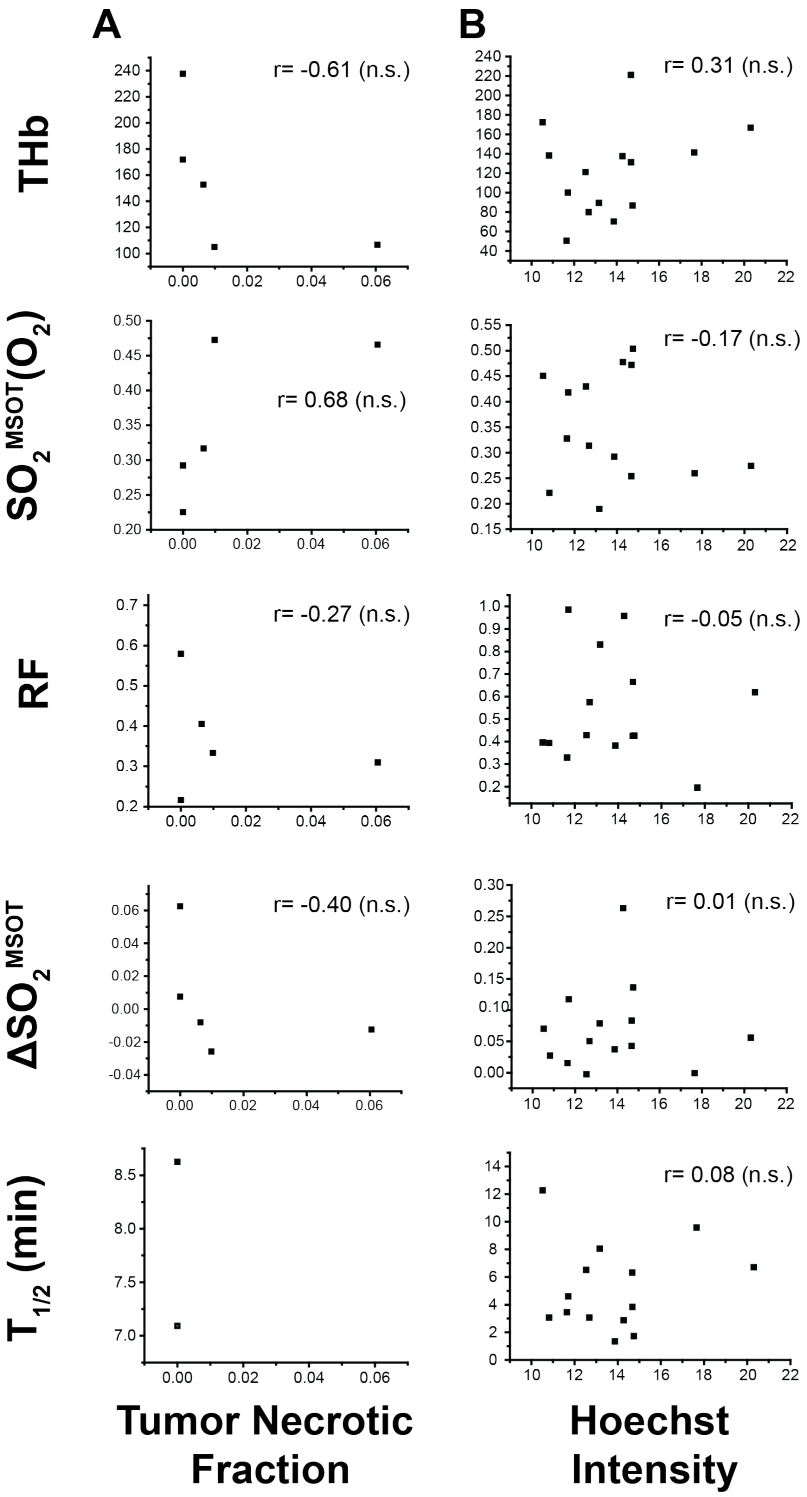

Figure S11: Relationship of mean OT and OE-OT metrics to tumour size. The graphs show changes in the OT metrics as tumours grow. For clarity, each line represents the change within one tumour between the first and last imaging sessions (meaning, first and second imaging session for animals sacrificed at the intermediate time point, or first and third imaging session for those sacrificed at the ethical approval limits). Significance level of the changes is indicated on each graph. * $p<0.05$, ** $p<0.01$, *** $p<0.001$.

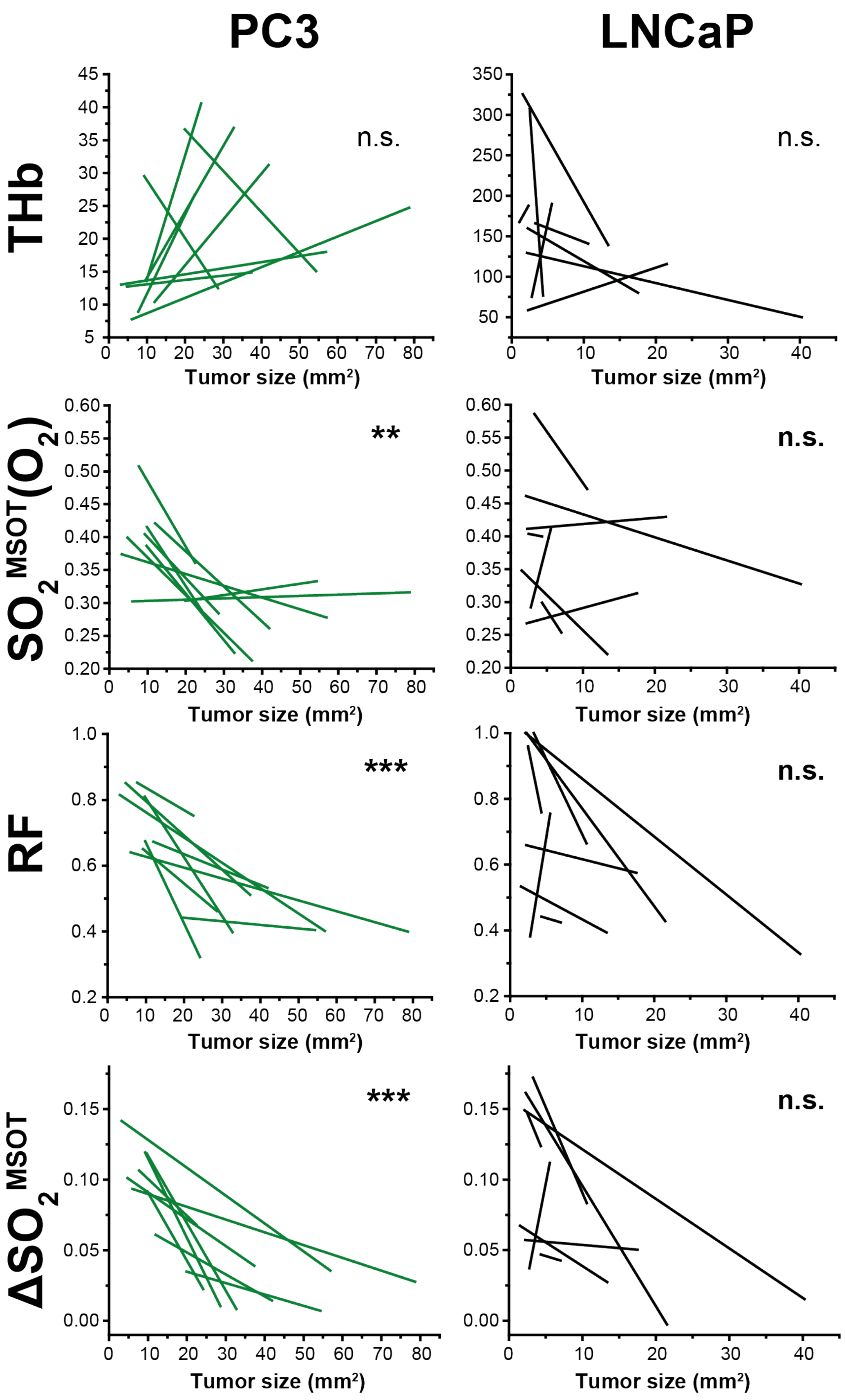

Supplement: Supplementary file 1 — Supplementary methods, figures and tables. [file thnov07p2900s1.pdf]
